# Method for Removing Spectral Contaminants to Improve Analysis of Raman Imaging Data

**DOI:** 10.1038/srep39891

**Published:** 2017-01-05

**Authors:** Xun Zhang, Sheng Chen, Zhe Ling, Xia Zhou, Da-Yong Ding, Yoon Soo Kim, Feng Xu

**Affiliations:** 1 Beijing Key Laboratory of Lignocellulosic Chemistry, Beijing Forestry University, Beijing, 100083, China; 2 Department of Wood Science and Engineering, Chonnam National University, Gwangju 500757, South Korea

## Abstract

The spectral contaminants are inevitable during micro-Raman measurements. A key challenge is how to remove them from the original imaging data, since they can distort further results of data analysis. Here, we propose a method named “automatic pre-processing method for Raman imaging data set (APRI)”, which includes the adaptive iteratively reweighted penalized least-squares (airPLS) algorithm and the principal component analysis (PCA). It eliminates the baseline drifts and cosmic spikes by using the spectral features themselves. The utility of APRI is illustrated by removing the spectral contaminants from a Raman imaging data set of a wood sample. In addition, APRI is computationally efficient, conceptually simple and potential to be extended to other methods of spectroscopy, such as infrared (IR), nuclear magnetic resonance (NMR), X-Ray Diffraction (XRD). With the help of our approach, a typical spectral analysis can be performed by a non-specialist user to obtain useful information from a spectroscopic imaging data set.

Using Raman imaging technique to obtain colourful chemical images is just a beginning for studying the chemical properties of a sample^1^. Chemists are more interested in interpreting the secrets locked within the spectral imaging data. Further data analysis, e.g. multivariate methods, allows the convoluted information content to be sorted according to the hypothesis that the original data is reconstructed from a limited number of significant factors^2^. However, such analysis is particularly sensitive to the presence of outliers, so that the original spectra involving spectral contaminants cannot be analyzed directly^3^.

Generally, there are two major contaminants that spill over into the Raman channels along with the actual signals: (1) sample and background fluorescence, as well as thermal fluctuations of the charge coupled device (CCD), can markedly affect the spectral baseline resulting in baseline drifts^4^; (2) cosmic rays are sporadic background artifacts detected by sensitive detectors, which manifest in spectra as narrow-bandwidth spikes^5^. These samples or instrument dependent contaminants are inevitable during the measurement. Therefore, it is essential that the spectral data should be pre-processed by commonly used methods, which are available in instrumentation software or analysis programs before other algorithms are implemented (Table S-1).

Methods for handling baseline drifts and spikes are two independent subjects. The diverse sources of background and additive noise make it hard to correct baseline for experimental spectral data^6^. Wavelet transform^7^, derivative^8^, robust local regression^9^ and polynomial fitting^10^ were introduced to eliminate the varying background. Some drawbacks, however, such as poor performances in low signal-to-noise ratio environments and dependence on a given user’s experience, have to be eliminated because they may lead to poor reproducibility of the calibration results. The approaches for removing spikes commonly fall into two categories depending on how the algorithm is designed. Methods in first category try to exclude the spikes on a single-scan spectrum via filtering algorithms such as wavelet processing, median filters and polynomial filters^11^. These methods suffer from serious limitations since they rely on an assumption of maximum spike bandwidth. Spectral distortion occurs when the bandwidth of spikes is comparable to spectral features of interest^12^. The alternative category suppresses spikes by comparing measured similar spectrum (referential spectrum). A typical example is “nearest neighbor correlation algorithm”, which firstly confirms a referential spectrum by comparing the cross-correlation coefficient between spectra of adjacent and secondly eliminates the spikes by setting a threshold value on the basis of the user’s experience^13^. The core of this category is how to confirm the referential spectrum, while the cross-correlation coefficient is inadequate to describe the features of numerical value. Moreover, the threshold value set by experience will also lead to unstable results. As a result, although this algorithm provides a new idea to resolve this issue, most of the applications are still limited to simulation data. Practical examples of this method are particularly scarce.

Here, we propose a novel approach for intelligently removing the spectral contaminants to improve analysis of Raman imaging data named “automatic pre-processing method for Raman imaging data set (APRI)” (Fig. 1). APRI consists of two complementary algorithms: (a) the adaptive iteratively reweighted penalized least-squares (airPLS algorithm) for baseline correction (Fig. 2a); (b) despiking algorithm on the basis of principal component analysis (PCA-despiking algorithm, Fig. 2b). Our method is neither dependent on the sample characteristics nor the measurement conditions. With the use of APRI, a typical spectral analysis can be performed by a non-specialist user to obtain useful information from a spectroscopic imaging data set.

## Methods

### Materials

An inclined 10-year-old poplar tree (*Populus nigra* L.) was provided by the arboretum of Beijing Forestry University, China. A small sample block was cut out from the seventh annual ring of the xylem. Without any embedding routing, a 10-μm-thick transverse section was prepared on a sliding microtome (Leica 2010R). It was then placed on a glass slide with a drop of D_2_O and sealed with a coverslip for micro-Raman measurement. In Raman spectrum, D_2_O can reduce the fluorescence of lignin and has a marked peak at 2490 cm^−1^.

### Micro-Raman system

The Raman imaging data set was acquired by using a micro-Raman system (LabRam Xplora, Horiba Jobin Yvon) equipped with a confocal microscope (Olympus BX51) and a motorized stage. The measurement was performed at room temperature (25 ± 3 °C). The entire optical system is diffraction-limited up to an object-side numerical aperture (NA) of 1.40 (Olympus, 100×, oil) and, consequently, is provided with a high spatial resolution in axial and lateral dimensions. Linear polarized laser (λ = 532 nm) was focused with a small spot size (theoretical 1.22λ/NA). Its power on the sample surface was around 8 mW. The Raman light was collected by a semiconductor-cooled charge coupled device (CCD) detector behind a grating spectrometer (1200 groves/mm). We selected 0.5 μm as the step for mapping and each pixel corresponds to one scan. The spectrum of each scan was obtained by averaging 4 s cycles. The confocal aperture was set at 100 μm. The reported depth resolution for the 100 μm confocal hole, based on the silicon (standard) phonon band at 520 cm^−1^, was ~ 4 μm.

### Data processing

The instrumentation software LabSpec (Horiba Jobin Yvon) was utilised to setup and control the micro-Raman system. The .ngc file, which is the format of the original Raman imaging data, was converted to .mat file for further data processing. Our software, APRI, is available as a Matlab script. The open-source code for APRI is available in Supplementary Materials.

### Baseline correction method: airPLS algorithm

Conventional notation was adopted throughout this paper: uppercase bold face letter for matrices (as **X**), lowercase boldface for vectors (as **X**), italicized subscript characters for vector index (as *x*_*i*_ or *z*_*i*_), and lowercase italicized letters for scalars (as *x*_*i*_(*j*)). Superscripts are assigned as follows: T, vector or matrix transpose; and −1, matrix inverse. A Raman imaging data can be regarded as a matrix **X** of dimension *m* by *n*, in which the digitized Raman spectrum of each recorded position corresponds to a row vector in the data table:


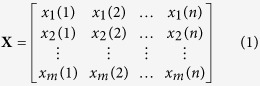


where *m* is the number of spectra traces in this data set and *n* is the number of data points per spectrum along the wavenumber (or any other spectral variable) axis, respectively.

The adaptive iteratively reweighted penalized least-squares (airPLS) algorithm was first proposed by Zhang Z.M. in 2010^14^. It works by treating the Raman imaging data set **X** as vectors in Hilbert space. We assume that **x** is the row vector of initial spectral data and **z** is the fitted vector. The fitted vector **z** is calculated by balancing the fidelity of **z** to **x** and the roughness of **z**:


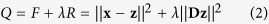


where *Q* is the balancing result, *F* is the fidelity expressed as the sum of squared errors between **x** and **z**, *R* is the roughness computed as the first derivative (matrix **D**) of **z**, *λ* is a parameter for controlling smoothness of the fitted vector (balancing coefficient), respectively. By finding the vector of partial derivatives and equating it to zero, i.e. ∂*Q*/∂**z** = 0, the solution of minimization problems of equation (2) is given as follows:


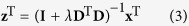


A weight vector of fidelity **W**, which is a diagonal matrix with *w*_*i*_ on its diagonal, is introduced for baseline correction. Thus equation (2) and equation (3) change to:


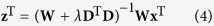


Without setting zeros to the weight vector at positions corresponding to peak segments, the equation (4) can be categorized as a smoothing algorithm. Subsequently, the adaptive iteratively reweighted procedure is performed to calculate the weights and adds a penalty item to control the smoothness of the fitted baseline. Each step of the adaptive iteratively reweighted procedure involves solving a weighted penalized least squares problem of the following form:


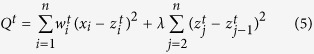


The weight vector **w** is obtained adaptively using an iterative method. One should give an initial value **w**^0^ = 1 at the starting step. After initialization, the **w** of each iterative step *t* can be obtained using the following expressions:


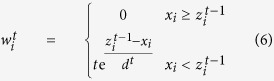


Vector *d*^*t*^ consists of negative elements of the differences between **x** and **z**^*t*−1^ in the *t*^th^ iteration step. The fitted value **z**^*t*−1^ in the previous (*t*−1)^th^ iteration is a candidate of baseline. If the value of the *i*^th^ point is greater than the candidate of baseline, it can be regarded as part of a peak. Thus its weight is set to zero to ignore this point at the next iteration of fitting. In airPLS algorithm, the iterative and reweight approaches are utilized to gradually and automatically eliminate the points of peaks and preserve the baseline points in the weight vector **w**. Iteration will stop either with the maximal iteration times or when the terminative criterion is reached. The terminative criterion is defined by:


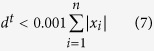


**Figure 3:**
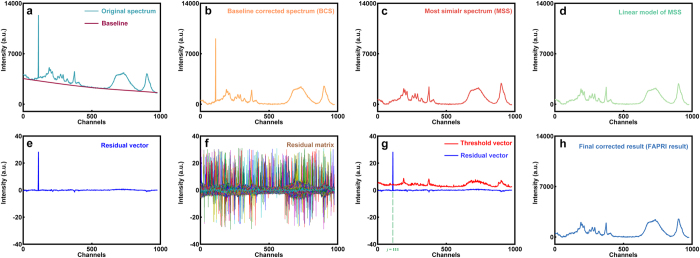
The procedure of APRI for a single spectrum. (**a**) The two spectral contaminants (baseline drift and spike) are observed in original spectrum. The baseline is calculated by using airPLS algorithm. (**b**) The baseline corrected spectrum (BCS) is corrected by subtracting the baseline from the original spectrum. (**c**) The most similar spectrum (MMS) is confirmed by PCA. (**d**) Linear fitting operation is necessary to reduce the large differences between BCS and MSS. Here, the spectrum of linear model is similar to that of MSS since the differences among BCS and MSS are not so apparent. (**e**) The standard residual vector is available by subtracting linear model of MSS from BCS. (**f**) The residual matrix is obtained by stacking the entire residual vectors together. This matrix is applied to determine the threshold vector. (**g**) The position of spike is located based on the residual vector and the threshold vector. (**h**) The two spectral contaminants are suppressed in the final pre-processed result.

The vector *d*^*t*^ also consists of negative elements of the differences between **x** and **z**^*t*−1^ (Fig. 3a). The corrected data x^*^ is achieved by subtracting finally fitted baseline **z** from original data **x** (Fig. 3b). Here, the maximum iterative time and *λ* are respectively set to 20 and 10^7^ by considering the computation time and the required smoothness of the corrected result. The original algorithm is open source software available in https://code.google.com/archive/p/airpls/downloads.

### Spikes removal method: PCA-despiking algorithm

Principle component analysis (PCA) is one of the multivariate methods and has been widely used with large multidimensional data sets^15^. The use of PCA allows the number of variables in a multivariate data set to be reduced, while retaining variation present in the data. The correlation coefficient array *R*_*j*_(*l*) of the baseline corrected data X^*^ is calculated as follows:


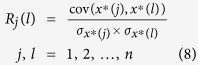


where cov(x^*^(*j*), x^*^(*l*)) is the covariance of column vectors x^*^(*j*) and x^*^(*l*), 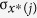 is the standard deviation of x^*^(*j*), and 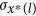 is the standard deviation of x^*^(*l*), respectively.

Then the eigenvalues and eigenvectors are determined from the correlation coefficient array,


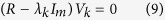


where *λk*—eigenvalues and 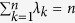; *V*_*k*_ = [*v*_*k*_(1), *v*_*k*_(2), …, *v*_*k*_(*n*)]^T^—eigenvectors corresponding to the eigenvalues *λ*_*k*_.

The eigenvectors are aligned in descending order with respect to their eigenvalues, i.e. *λ*_*k*_ > *λ*_*k*+1_. The uncorrelated principal component is formulated as:


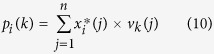



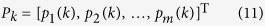


where *P*_*k*_ is termed as the principal component. When applying PCA to the data, a significant factor is how many principal components to keep. This is determined by using the proportion of total population variance. The proportion (*PR*) of *k*^th^ principal component (*P*_*k*_) is shown as follows:


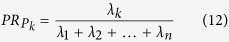


where *λ*_*k*_ is the eigenvalue (variance) of *k*^th^ eigenvector. This represents the explanation of *k*^th^ principal component for the spectral data set. In APRI, the first *q* principal components are retained when the cumulative *PR* exceeds 85%, which means that 85% of the data variance was explained. The selected principal components can reflect the original data very well and thus the dimension of the data is reduced to save the running time in subsequent calculation of the distances. A principal components matrix *PC* (dimension *m* by *q*) is then achieved. In this matrix, each row of *PC* is treated as the feature of corresponding Raman spectrum.

The squared Euclidean distance array *D*_*i*_(*j*) between each two features is calculated as:


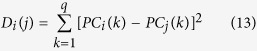


where *PC*_*i*_(*k*) and *PC*_*j*_(*k*) are the features of *i*^th^ and *j*^th^ spectrum in *k*^th^ principal components. The most similar spectrum (MSS, 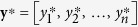) of each spectrum (**y** = [*y*_1_, *y*_2_, …, *y*_*n*_]) is determined by finding the minimal Euclidean distance between the features of baseline corrected spectrum (BCS) and referential spectra (Fig. 3c). Since all of the spectra are taken into consideration, it should be noted that the procedure of confirming MSS will cost a lot of time if the size of original data set is too large.

A linear model is proposed by the least-squares algorithm to further reduce the variation between **y** and y* (Fig. 3d). The linear regression spectrum **y**_*re*_ is generated by y*, and the sum squares errors *S* between **y** and **y**_*re*_ is given as follows:


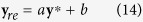



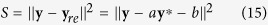


where *a* is the scaling factor and *b* is the offset, respectively. By calculating the vector of partial derivatives and equating it to zero, i.e. ∂*S*/∂*a* = 0, ∂*S*/∂*b* = 0, the solution of *a* and *b* is achieved,


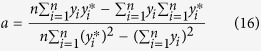



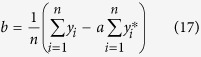


where *y*_*i*_ and 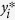 are elements of **y** and y*, respectively. The residual vector **e** is calculated as follows:


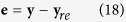


**Figure 4:**
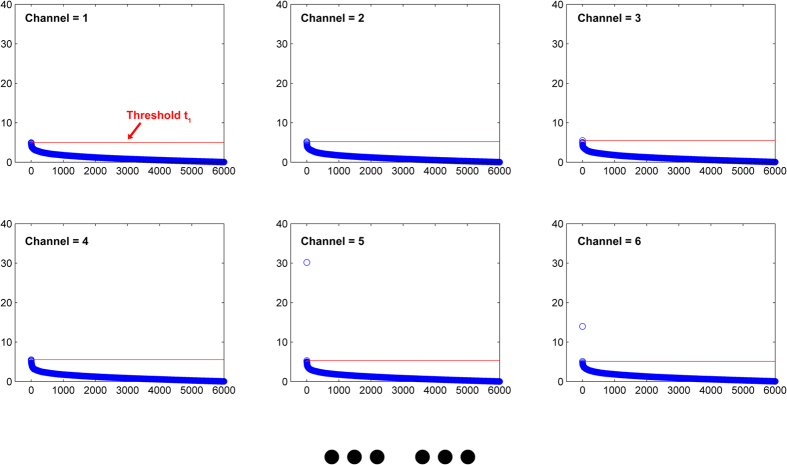
Automatic generation of the threshold vector t. For channel *j* of the new residual vector **E**, we just consider the values that are positive based on the positive nature (unidirection) of spikes. Threshold **t**_*j*_ should satisfy that **t**_*i*_ = **E**_*ij*_, (***d*****E**_*j*_ < −1).

This procedure is applied to magnify the intensity of existing spikes and makes them more obvious targets for locking their positions (Fig. 3e). The residual vector **e** is normalized by:


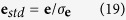


where *σ*_**e**_ is the standard deviation of **e**. The normalization can ensure that the residual vector is independent of the numerical range of the spectral intensities. It is particularly important for extending our method to other samples. The determination of spike positions is based on a residual matrix **E**_0_ achieved by stacking all of **e**_*std*_ together (Fig. 3f). A threshold vector is required for filtering the spike signals in *E*_0_. Since the spike has features including narrow-bandwidth, relative high intensity, positive (unidirection) and random occurrences in spectral data, the spike signals in each channel should be positive numbers and far away from normal signals. We first obtain a new residual matrix **E** by ranking the values of each column in **E**_0_ are ranked from largest to smallest. The threshold vector **t** is then automatically generated at the point of abrupt change in each channel based on the derivative of **E** (Figs 3g and 4). The shape and width of spikes depend heavily on instrument/measurement mode and manufacturer software. In our experiment, a spike zone generally contains 20 to 40 variables in spectrum. Thus we select the spike range as 41 variables to ensure that all affected variables are included. Without consideration of the shape of the spike, the PCA-despiking algorithm is finished by replacing the spike zone with the corresponding **y**_*re*_ (Fig. 3h). It should be noted that a wider spike range means more variables are corrected, while introducing MSS can guarantee the shape of corrected spectrum shape won’t be distorted much. According to the principle of the algorithm, bad replacement may appear in final result if the spike zone of corresponding MSS also has spike noise. In this case, instead of the first referential spectrum, the spectrum with next minimal distance as the MSS is selected.

Our algorithm relies on the differences between BCS and MSS, which may lead to unsatisfactory results when the spikes of them appear at the same position. Although the probability of this event is low, it limits the performance of the algorithm to a certain extent. Since the MSS of each spectrum changes dynamically during the iterative procedure so that the appearances of spikes at the same position are evitable, our solution is to perform the APRI once again.

## Results and Discussion

**Figure 5:**
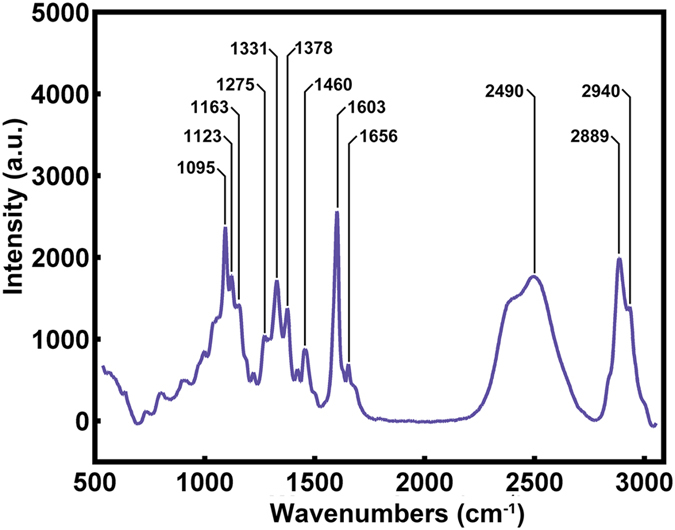
Typical Raman spectrum of poplar in presence of D2O, D2O can reduce the fluorescence of lignin and has a marked peak at 2490 cm^−1^.

Raman spectroscopy can provide chemical information about organic and inorganic substances quickly and nondestructively with little to no sample preparation. The analysis of organic compounds is much more difficult and complicated than that of inorganic ones. In this study, the experiments are performed on a transverse section of poplar xylem (a biological sample) to verify the validity of our method. The poplar cell wall is organized in several layers formed at different periods during cell differentiation with different proportions of components, including cellulose, hemicellulose and lignin. Figure 5 shows a typical Raman spectrum of poplar and its band assignments are displayed in Table 1 on the basis of previous literature^16^,^17^. As a typical biological material, the vibrational spectra of poplar are rather complex with overlapping bands. In particular, cellulose and hemicellulose are hard to discern in spectrum because of their similar chemical bonds^18^.

**Figure 6:**
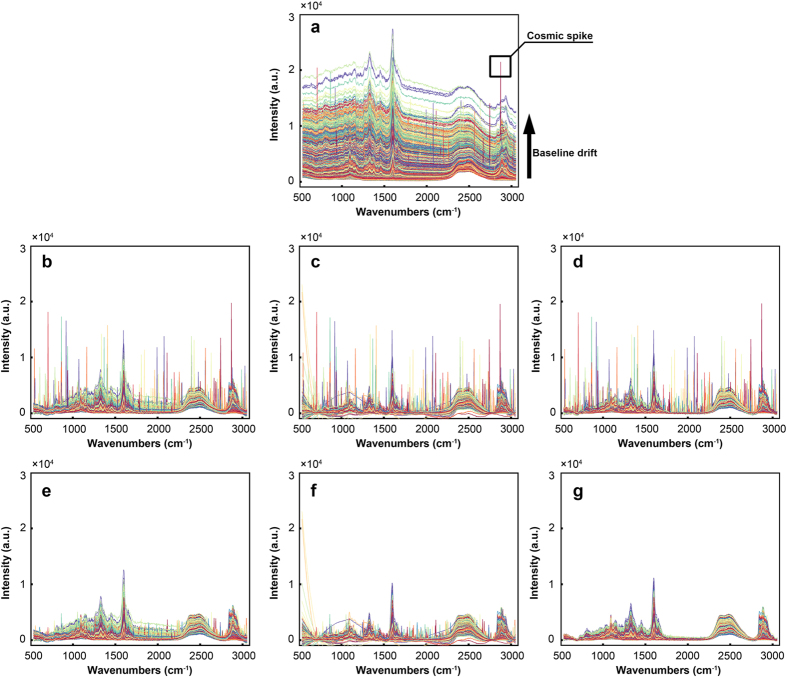
Data pre-processing results achieved by instrumentation software and APRI. (**a**) The plots of the original Raman imaging data. (**b**) Baseline correction by polynomial fitting (degree = 2). (**c**) Baseline correction by polynomial fitting (degree = 2). (**d**) Baseline correction by APRI (degree = 2). (**e**) Despiking operation by median filtering on the basis of (**b**). (**f**) Despiking operation by median filtering on the basis of (**c**). (**g**) Despiking operation by APRI on the basis of (**d**).

We collected 12,870 spectra from a 65 × 49.5 μm region with a spatial resolution 0.5 μm/pixel. It can be regarded as a matrix of 12,870 by 977 dimensions. The plots of original Raman spectral data are shown in Fig. 6a. We notice that the original data have significant baseline drifts and spikes. As previously mentioned, these spectral contaminants are caused by sample fluorescence and CCD. In plants, lignin is known to exhibit fluorescence under laser with specific wavelength, which interferes strongly with the determination of spectral peaks^19^. Many efforts have been undertaken by predecessors to eliminate these interferences, such as using near-infrared laser, but the problems are still considerable^20^.

The common used instrumentation software, such as LabSpec (Horiba Jobin Yvon) and OPUS (Bruker), provides the polynomial fitting algorithm and the median filtering algorithm to cope with the baseline drifts and the spikes problems, respectively. Polynomial fitting algorithm attempts to estimate the unknown background and abolish the sloped or oscillatory baselines based on user-defined polynomial degree and points^21^. However, it suffers from a very loose baseline if the degree of polynomial is not set properly. In addition, the unknown background of the spectrum is often too complicated to be fitted by a simple polynomial function in practice thereby rendering unsatisfactory corrected results. As shown in Figs 5c and 6b, changing the degree of polynomial will achieve different correction results. When the degree is set to 2, it is found that the correction of baseline is incomplete. If we enlarge the degree number to 8, significantly spectral distortion are observed, such as appearance of negative parts and changes in spectral shape. The median filtering algorithm requires the user to specify the maximum number of spike bandwidth and height as the references for determination of spikes, indicating that the algorithm is effective in detecting the sharp or high spikes but is unable to remove the spikes with low intensity^11^. Figure 5f and 6e show that the median filtering algorithm only can remove parts of the spikes.

The principle of APRI is fundamentally different, allowing automated operation by the applications of airPLS and PCA-despiking algorithm. The airPLS algorithm works by iteratively changing the weights of fitted spectrum to confirm the baseline function. Compared to the conventionally used algorithms of baseline correction, airPLS algorithm achieves a smoother result without adjustable parameters because the baseline is determined by balancing fidelity and roughness of the fitted spectrum. The PCA-despiking algorithm performs PCA to extract the main features (i.e. scores) of the baseline corrected spectra (BCS). For each BCS in the spectral data, the algorithm searches the most similar spectrum (MSS) from all the feature signatures (using the Euclidean distance) and excludes the spikes by comparing the differences between the BCS and MSS. The final results show that the spectral contaminants are perfectly removed (Fig. 6d and g). In order to prove that the results were not idiosyncratic to the particular Raman data set, other examples pre-processed by APRI can be found in Supplementary Information (Figures S-1 to S-5). The core of spike corrections is how to confirm the MSS. Here, we employ PCA and Euclidean distance to find out the MSS. Other judgment methods, such as Mahalanobis distance or Pearson correlation coefficient, also can be applied to determine the MSS. Mahalanobis distance is similar to our method (PCA plus Euclidean-Distance): both of them take into account the correlations of the data. The calculated amount of our method is less than Mahalanobis distance because the original data has been condensed by PCA. Using Pearson correlation coefficient, by contrast, requires only a low calculation cost. However, it is independent of scaling and thus may distort the final corrected spectra.

To further analyze the Raman spectral data, a standard PCA is performed on original and corrected data. The first 20 row vectors (loadings) of PCA results were selected to generate their mapping images (Figures S-6 to S-25). For the original data results, the baseline drifts exist in the 1–9 spectra with high spatial resolved mapping profiles, while the spikes are visible in the 11–20 spectra with low image resolution. This means that the PCA not only can extract and condense the features of contaminants in the Raman imaging data set, but also can be potentially used as an evaluation of the correction quality. It is found that both the baseline drifts and the spikes disappear in the PCA results of APRI corrected data. Moreover, the mapping profiles show a higher spatial resolution than the original ones, which suggests that the loadings of corrected data are more representative. Although APRI is a powerful tool for removing the spectral contaminants, it may not be suitable in certain applications. Our algorithm for spike correction depends crucially on the availability of a closely similar spectrum without spike at the same position. Spectral distortion may occur when the sample size is too small (generally spectral amounts less than 10, depending on the measurements). Large differences will be found between BCS and MSS thereby providing incorrect determination of the spikes. In such case, filtering algorithms that exclude the spikes on a single-scan spectrum is more appropriate. Additionally, APRI is computationally faster for large-scale Raman imaging data. It takes less than 4 minutes to correct 10,000 spectra on a standard desktop computer (Win7 OS, Intel Core i5-3.2Ghz CPU and 8GB RAM).

The spectral contaminants induced by sample or instrumental perturbations are not only common in Raman spectroscopy but also in other methods of spectroscopy, such as infrared (IR), nuclear magnetic resonance (NMR), X-Ray diffraction (XRD). More information can be gleaned from the spectral data by applying various multivariate methods, whilst the unknown background or unwanted peaks may complicate the subsequent analysis of their data. The uniqueness of APRI is the self-adaptive nature of airPLS and PCA-despiking algorithms, which removes the contaminants on the basis of the spectral features themselves. Therefore, APRI is potential to be an effective, freely available tool for pre-processing the data of other spectroscopic techniques to improve the quality of data analysis.

## Additional Information

**How to cite this article**: Zhang, X. *et al*. Method for Removing Spectral Contaminants to Improve Analysis of Raman Imaging Data. *Sci. Rep.* **7**, 39891; doi: 10.1038/srep39891 (2017).

**Publisher's note:** Springer Nature remains neutral with regard to jurisdictional claims in published maps and institutional affiliations.

## Supplementary Material

Supplementary Information

## Figures and Tables

**Figure 1:**
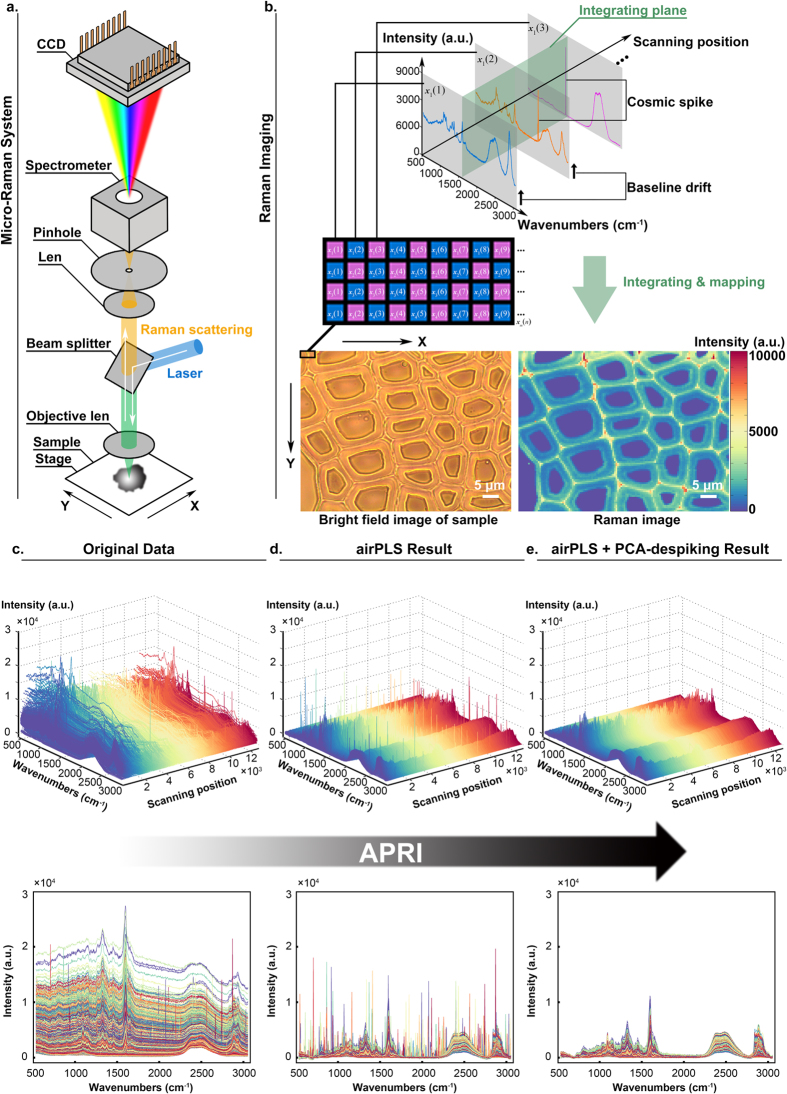
An overview of Raman imaging and APRI: The sample is measured by a micro-Raman system which couples an optical microscope to a Raman high-resolution spectrometer with a charge coupled device (CCD) detector. The bright field image obtained by the optical microscope is used to record the spatial information of the sample. The spectra are recorded as a matrix and corrupted by the contaminants from sample and CCD. The Raman image is achieved by integrating a specific Raman peak. Here we set a integrating plane around 1600 cm^−1^ to generate the Raman image of lignin. It can be used to locate the lignin semi-quantitatively.

**Figure 2:**
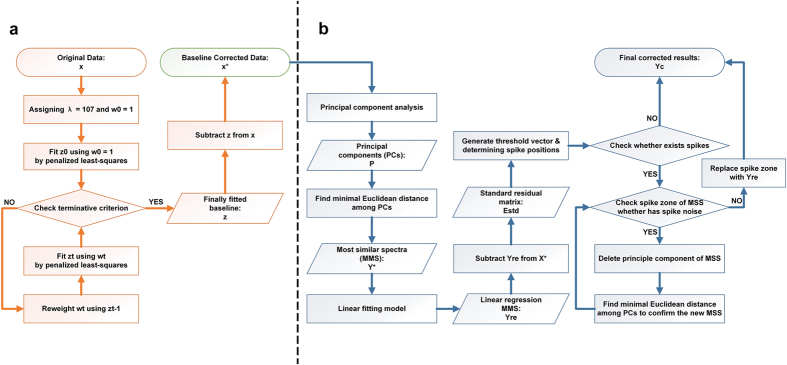
The flow chart of APRI: (**a**) adaptive iteratively reweighted penalized least-squares (airPLS) algorithm for baseline correction; (**b**) a despiking algorithm on the basis of principal component analysis (PCA-based despiking). Each step is corresponding to the equation in the Theory section.

**Table 1:** Raman peak positions and bands assignments for major structures of poplar.

Wavenumbers (cm^−1^)	Components	Assignments
1095	C, H	heavy atom CC and CO stretching vibration
1123	C, H	heavy atom CC and CO stretching vibration
1163	C, H	heavy atom CC and CO stretching vibration plus HCC and HCO bending vibration
1275	L	aryl-O of aryl OH and aryl O−CH3; guaiacyl ring with C=O group
1331	L, C, H	HCC and HCO bending vibration
1378	C, H	HCC, HCO, and HOC bending vibration
1460	L, C, H	HCH and HOC bending vibration
1603	L	aryl ring stretching vibration, symmetrical vibration
1656	L	ring conjugated C=C stretching vibration of coniferyl alcohol; C=O stretching vibration of coniferaldehyde
2889	C, H	CH and CH_2_ stretching vibration
2940	L, C, H	CH stretching vibration in OCH_3_ asymmetric vibration

C: Cellulose; H: Hemicellulose; L: Lignin.
