# Supplementary material for: Method for Removing Spectral Contaminants to Improve Analysis of Raman Imaging Data
Source: Sci Rep. 2017 Jan 5;7:39891. doi: 10.1038/srep39891 (PMC5215229; doi:10.1038/srep39891)
Supplement: Supplementary Information [file srep39891-s1.pdf]

SUPPLEMENTARY INFORMATION

Method for Removing Spectral Contaminants to Improve Analysis of Raman Imaging Data

Xun Zhang,[†] **Sheng Chen,**[†] **Zhe Ling,**[†] **Xia Zhou,**[†] **Da-Yong Ding,**[†] **, Yoon Soo Kim,**[‡] and **Feng Xu**[*†]

[†]Beijing Key Laboratory of Lignocellulosic Chemistry, Beijing Forestry University, Beijing, 100083, China

[‡]Department of Wood Science and Engineering, Chonnam National University, Gwangju 500757, South Korea*

Email: xfx315@bjfu.edu.cn. Tel/Fax: +86-10-62337993

Table S-1 Common data pre-processing software for Raman spectral data sets.

Software	Website	Description	License
LabSpec	http://www.horiba.com/	Instrumentation software of Horiba products	Commercial
WiRE	http://www.renishaw.com/en	Instrumentation software of Renishaw products	Commercial
OPUS	http://www.bruker.com/	Instrumentation software of Bruker products	Commercial
CytoSpec	http://www.cytospec.com/ftir.php	Analysis of vibrational spectroscopic (IR and Raman) imaging data sets	Commercial
ImageLab	http://www.imagelab.at/en_home.html	Multisensor imaging tool for processing and analysing hyperspectral images	Commercial
Matlab	http://www.mathworks.com/products/matlab/	Platform for machine learning, signal processing, image processing, computer vision and much more	Commercial
Extended Multiplicative Signal Correction (EMSC) Toolbox	http://www.models.life.ku.dk/emsctoolbox	EMSC for spectral interference subtraction and extended inverted scatter correction	Open source
Biodata Toolbox	http://www.mathworks.com/matlabcentral/fileexchange/22068-biodata-toolbox	Database system coupled to chemometrics that consequently stores spectra and related data	Open source
IRootLab	http://trevisanj.github.io/irootlab/	Toolbox for vibrational spectroscopy data analysis	Open source
Multivariate Image Analysis (MIA) Toolbox	http://www.eigenvector.com/software/mia_toolbox.htm	Hyperspectral image analysis based on PLS_Toolbox	Open source
Multivariate Curve Resolution-Alternating Least Squares (MCR-ALS) Toolbox	http://www.cid.csic.es/homes/rtaqam/tmp/WEB_MCR/welcome.htm	Tool for multivariate curve resolution	Open source
Raman Processing Program	http://cares.wayne.edu/rp/	Tool for processing, analysing and classifying Raman spectra	Open source
Origin for Spectroscopy	http://www.originlab.com/index.aspx?go=Solutions/Applications/Spectroscopy	Software for analysing spectral data	Commercial
PeakFit	https://systatsoftware.com/products/peakfit/	Automatic method for handling peaks in spectroscopy, chromatography and electrophoresis	Commercial
The Unscrambler X	http://www.camo.com/rt/Products/Unscrambler/unscrambler.html	Software set the standard in multivariate methods for quickly, easily and accurately analysing the experimental data	Commercial

Figure S-1

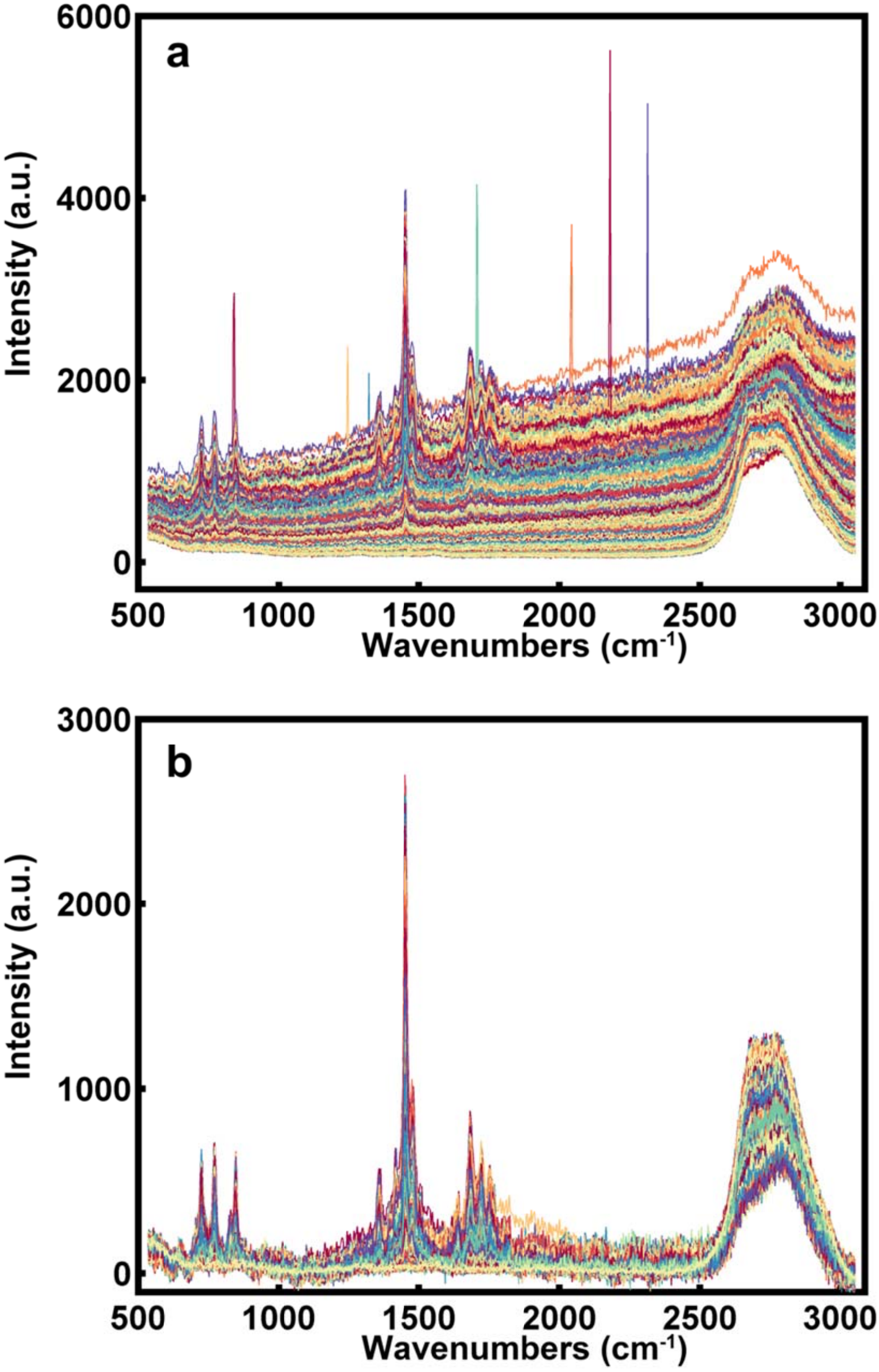

More examples of Raman imaging data set pre-processed by APRI: (a) Original Raman spectra; (b) Raman spectra pre-processed by APRI. (Material: Pulp**. Spectral amounts:** 616**).**

Figure S-2

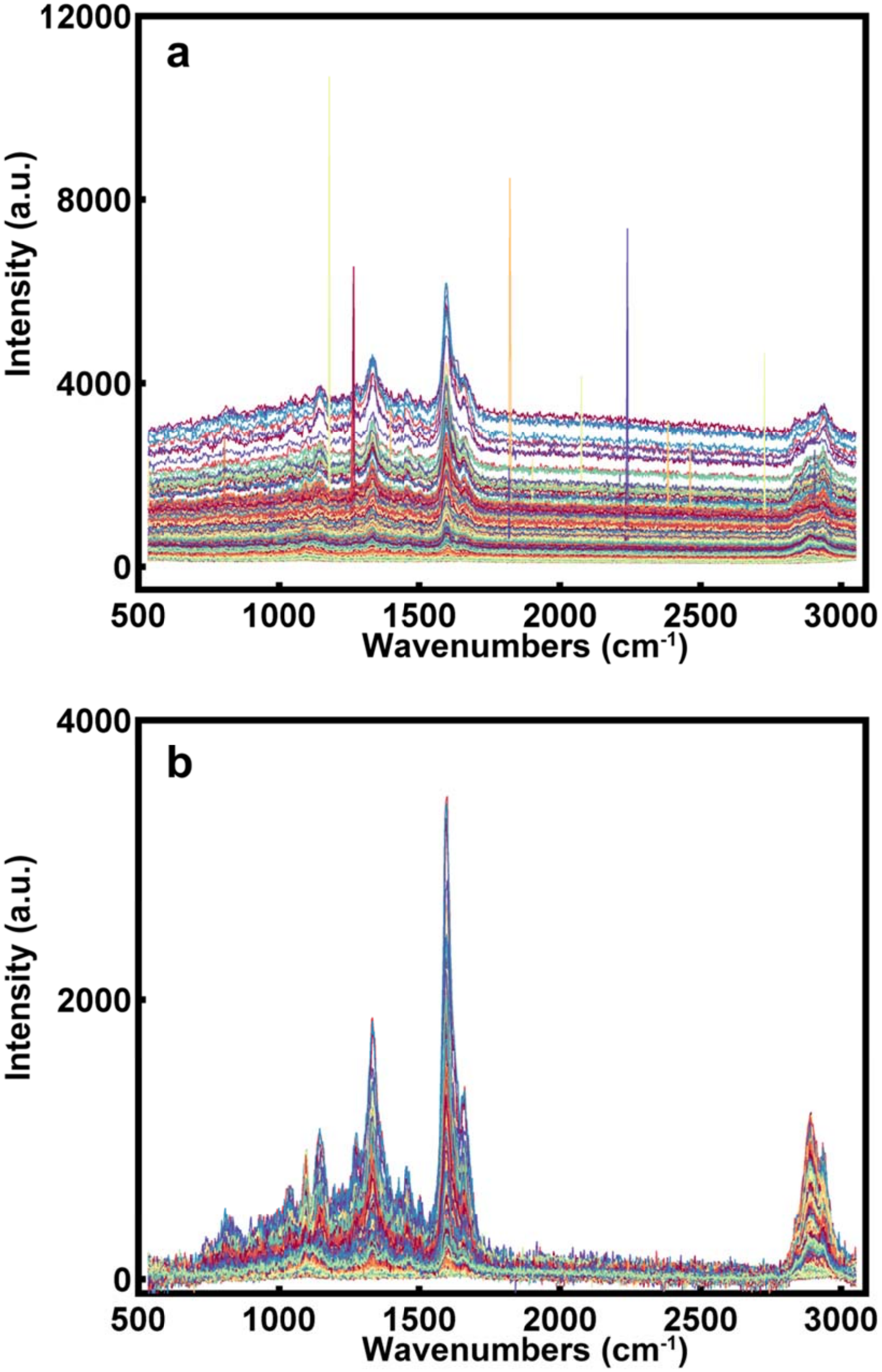

More examples of Raman imaging data set pre-processed by APRI: (a) Original Raman spectra; (b) Raman spectra pre-processed by APRI. (Material: Poplar**. Spectral amounts:** 1024**).**

Figure S-3

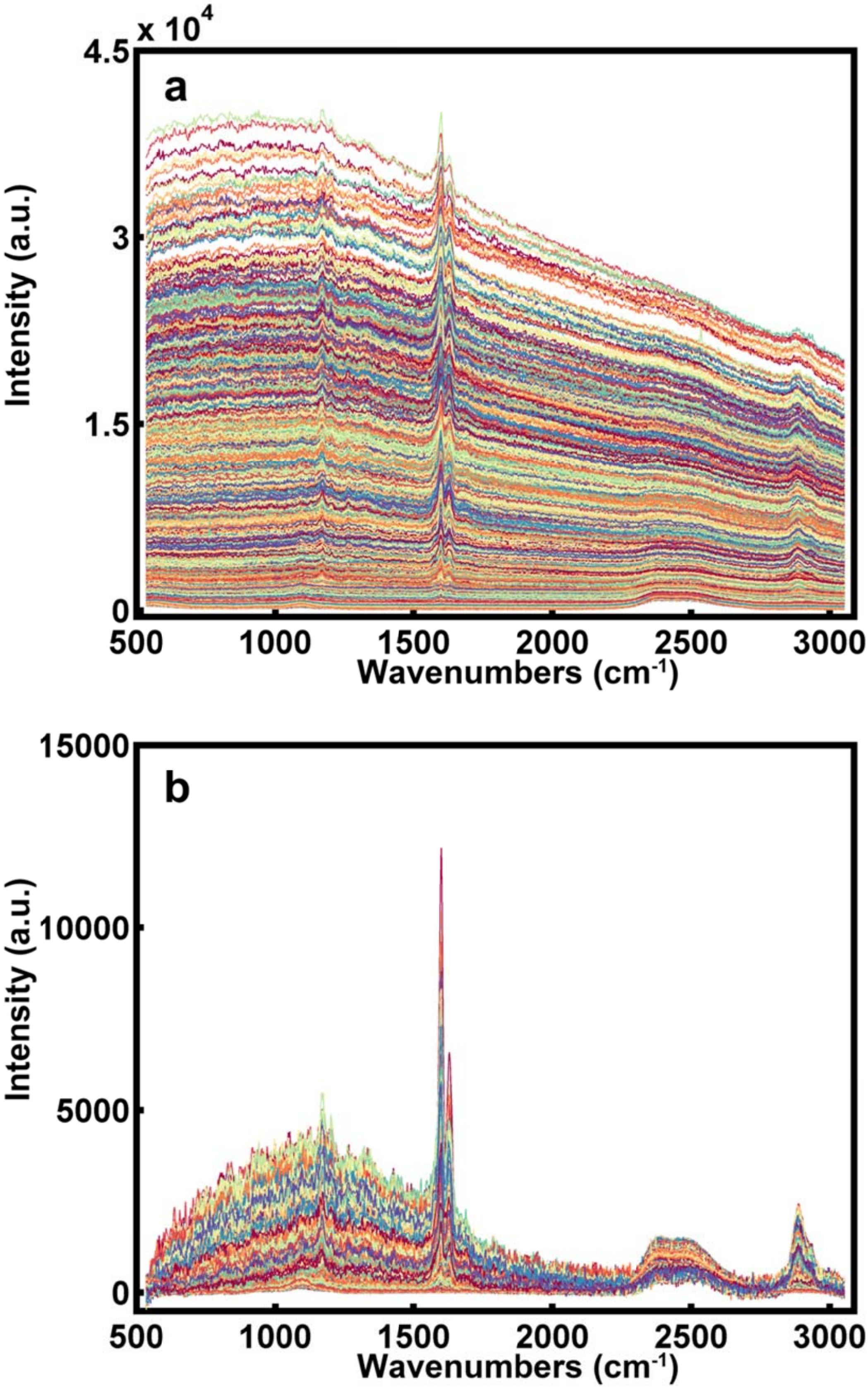

More examples of Raman imaging data set pre-processed by APRI: (a) Original Raman spectra; (b) Raman spectra pre-processed by APRI. (Material: *Miscanthus sinensis***. Spectral amounts:** 616**).**

Figure S-4

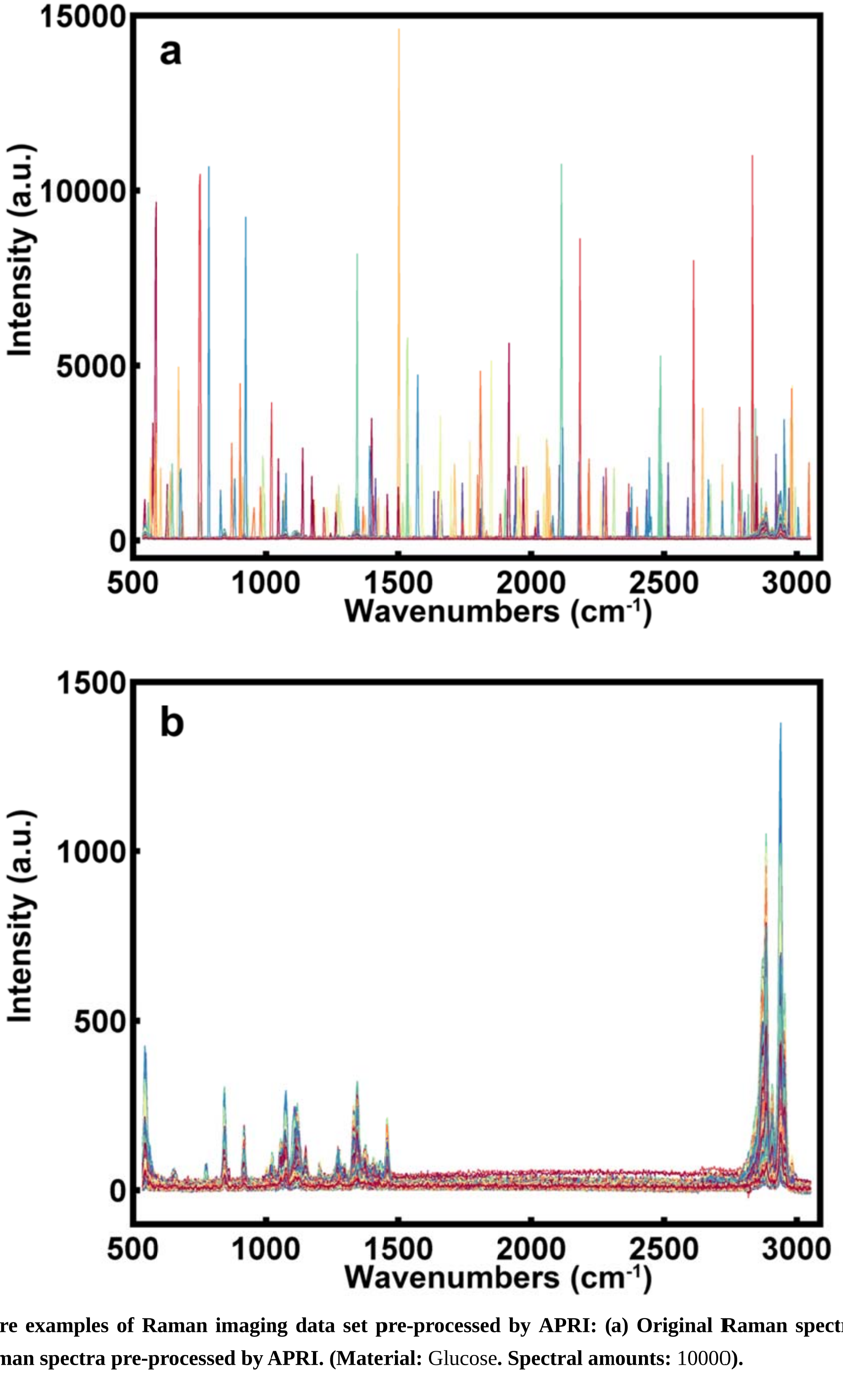

More examples of Raman imaging data set pre-processed by APRI: (a) Original Raman spectra; (b) Raman spectra pre-processed by APRI. (Material: Glucose**. Spectral amounts:** 10000**).**

Figure S-5

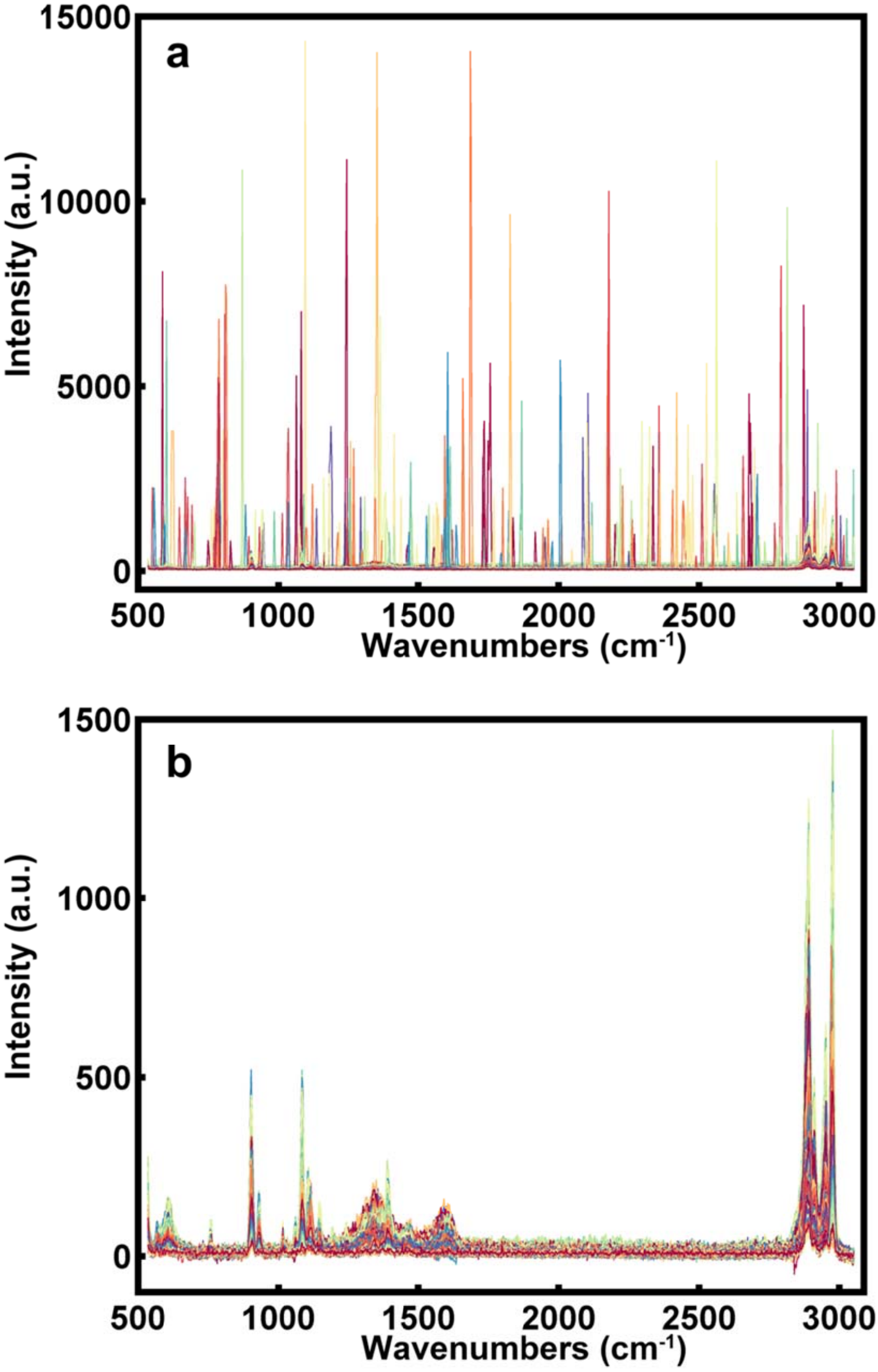

More examples of Raman imaging data set pre-processed by APRI: (a) Original Raman spectra; (b) Raman spectra pre-processed by APRI. (Material: Xylose**. Spectral amounts:** 10000**).**

Figure S-6

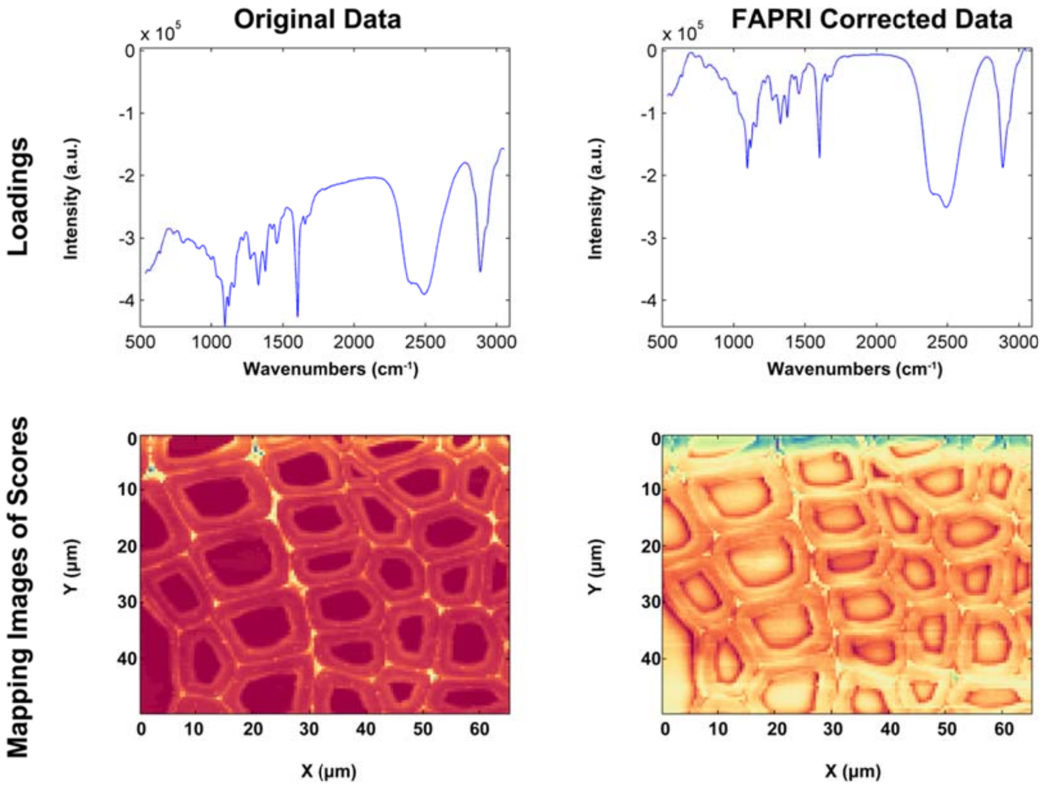

PCA results of original data and APRI corrected data (The 1st loading).

Figure S-7

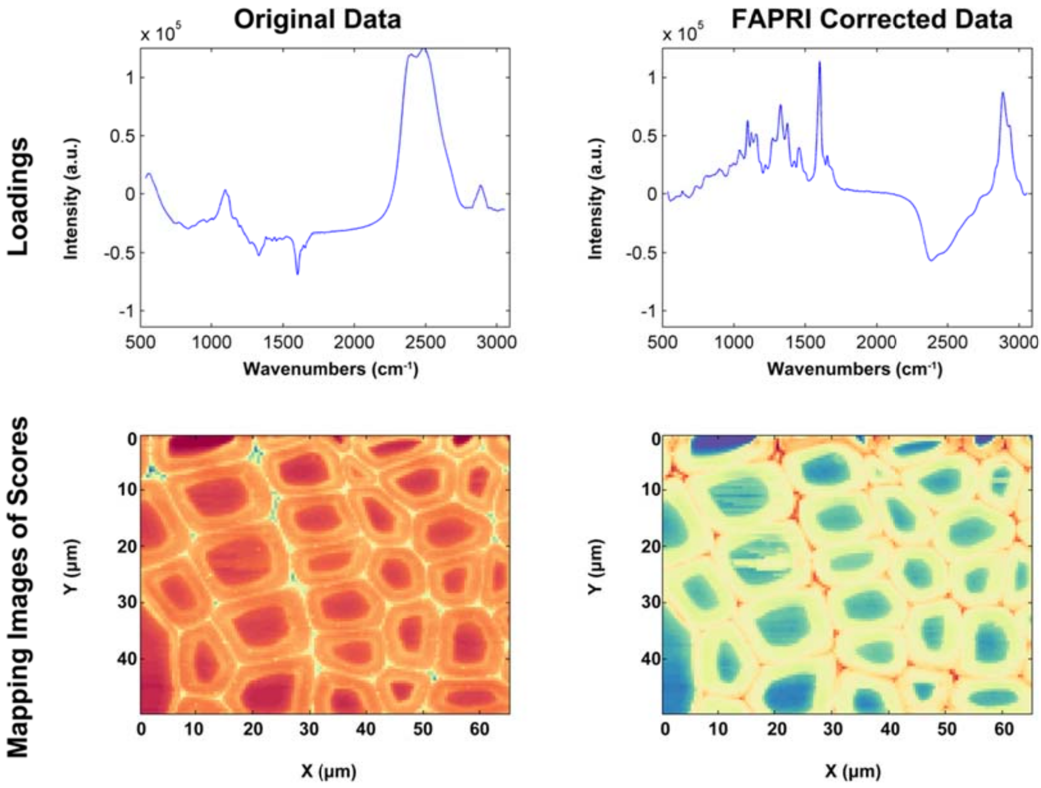

PCA results of original data and APRI corrected data (The 2nd loading).

Figure S-8

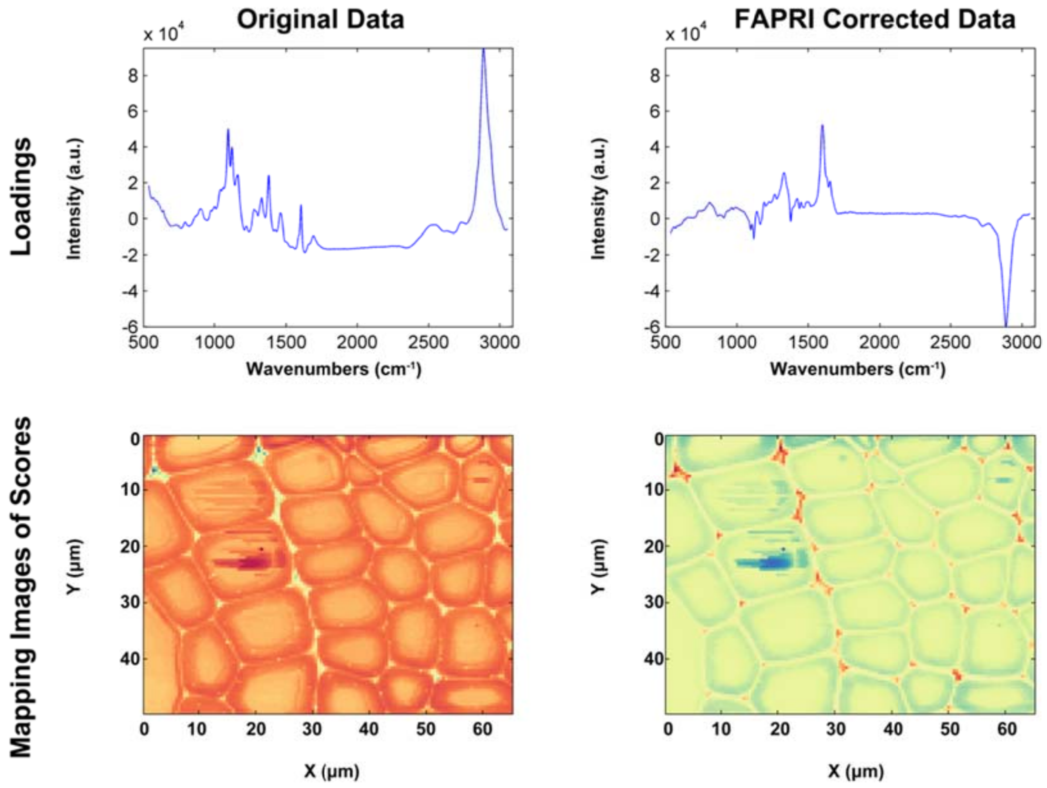

PCA results of original data and APRI corrected data (The 3rd loading).

Figure S-9

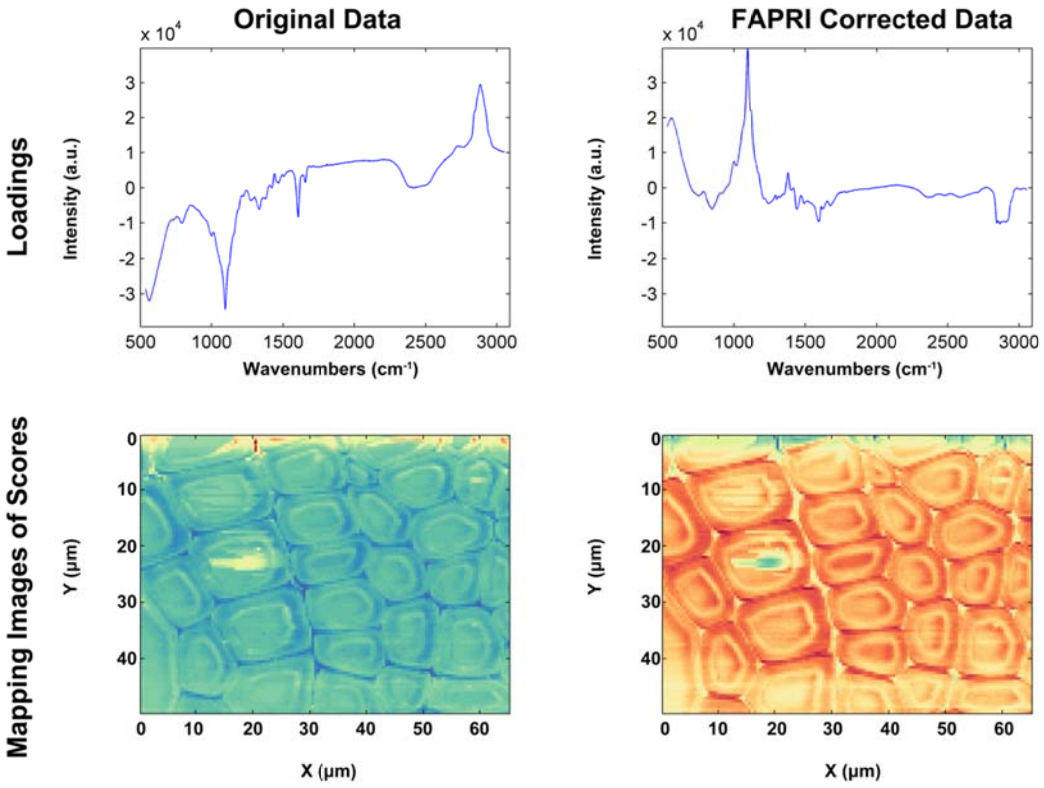

PCA results of original data and APRI corrected data (The 4th loading).

Figure S-10

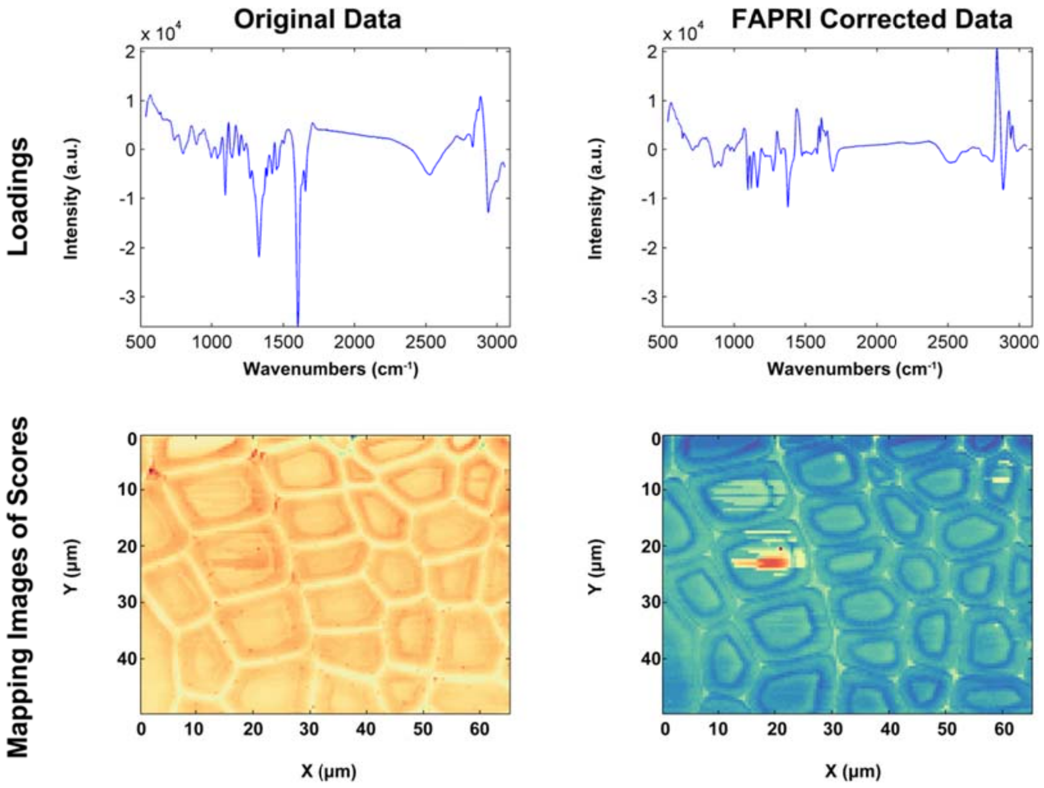

PCA results of original data and APRI corrected data (The 5th loading).

Figure S-11

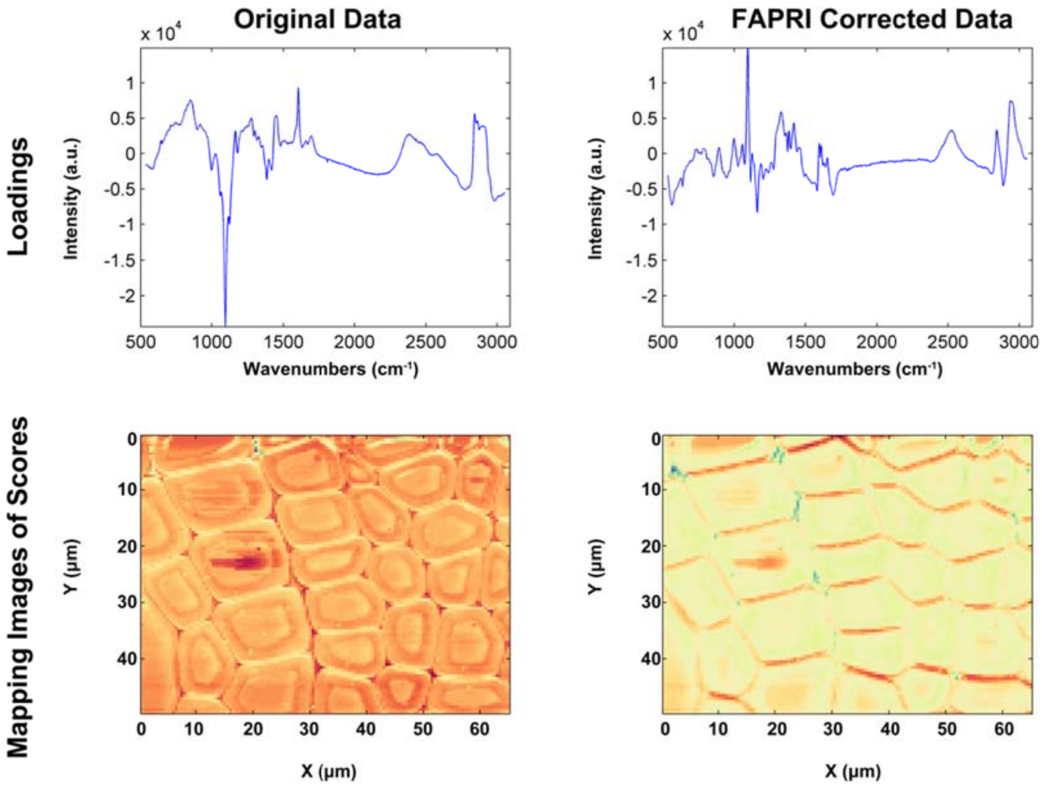

PCA results of original data and APRI corrected data (The 6th loading).

Figure S-12

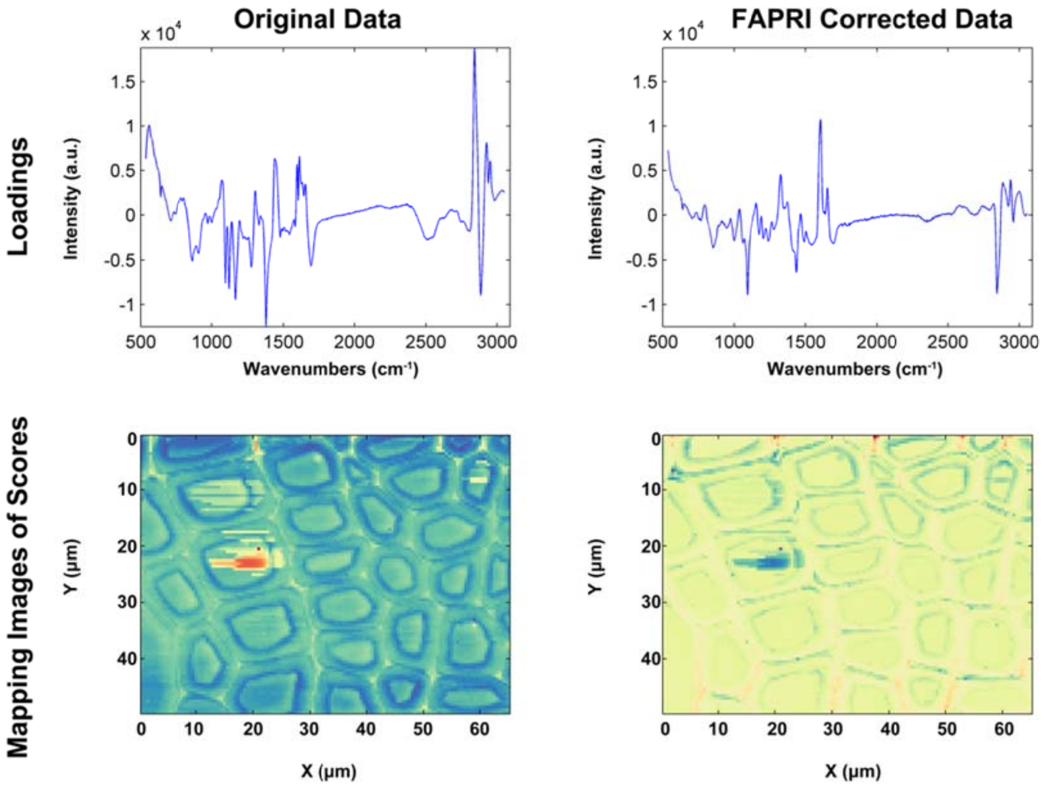

PCA results of original data and APRI corrected data (The 7th loading).

Figure S-13

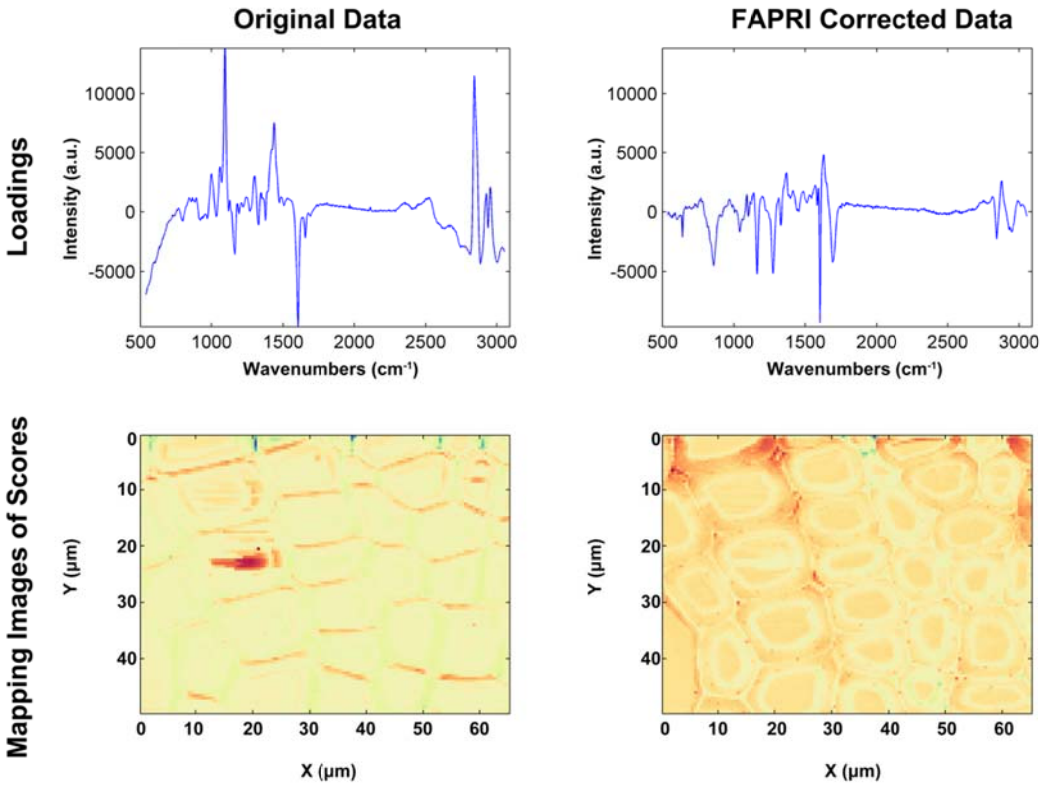

PCA results of original data and APRI corrected data (The 8th loading).

Figure S-14

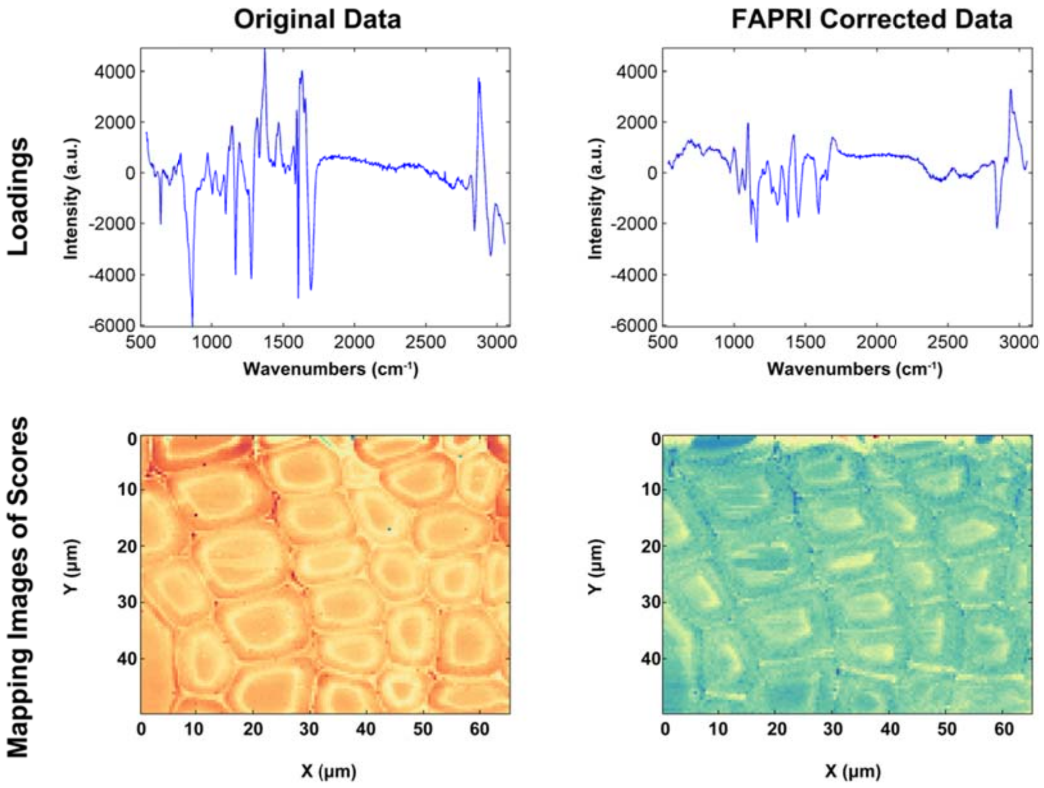

PCA results of original data and APRI corrected data (The 9th loading).

Figure S-15

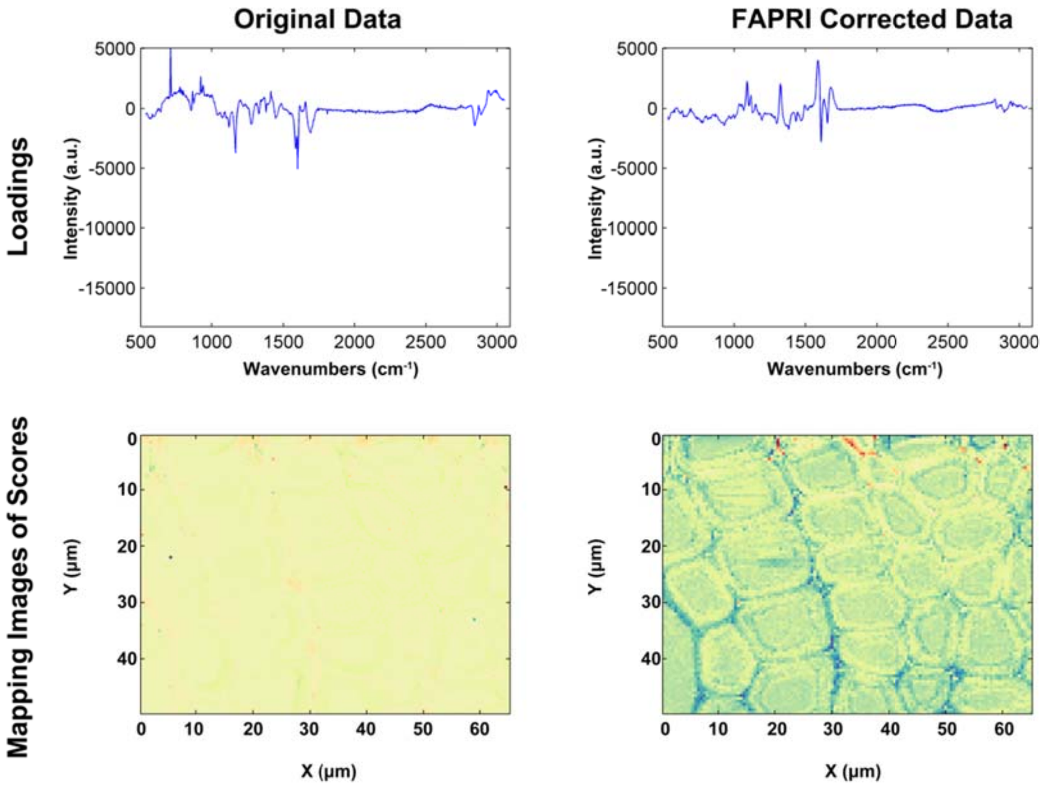

PCA results of original data and APRI corrected data (The 10th loading).

Figure S-16

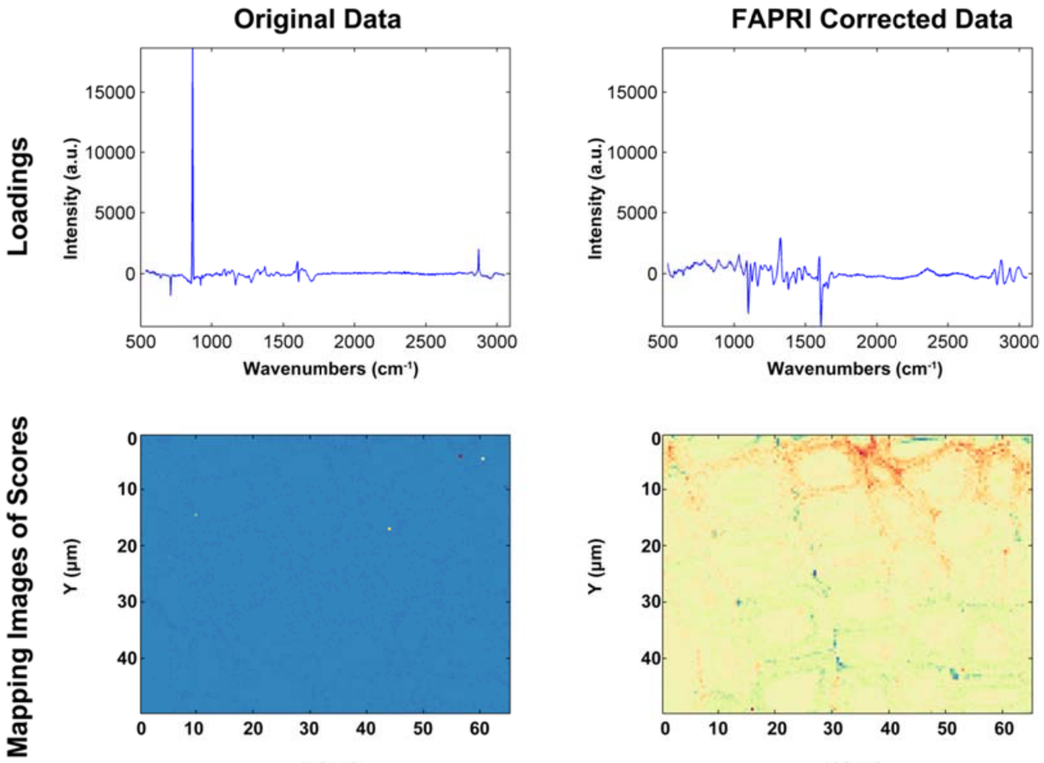

PCA results of original data and APRI corrected data (The 11th loading).

Figure S-17

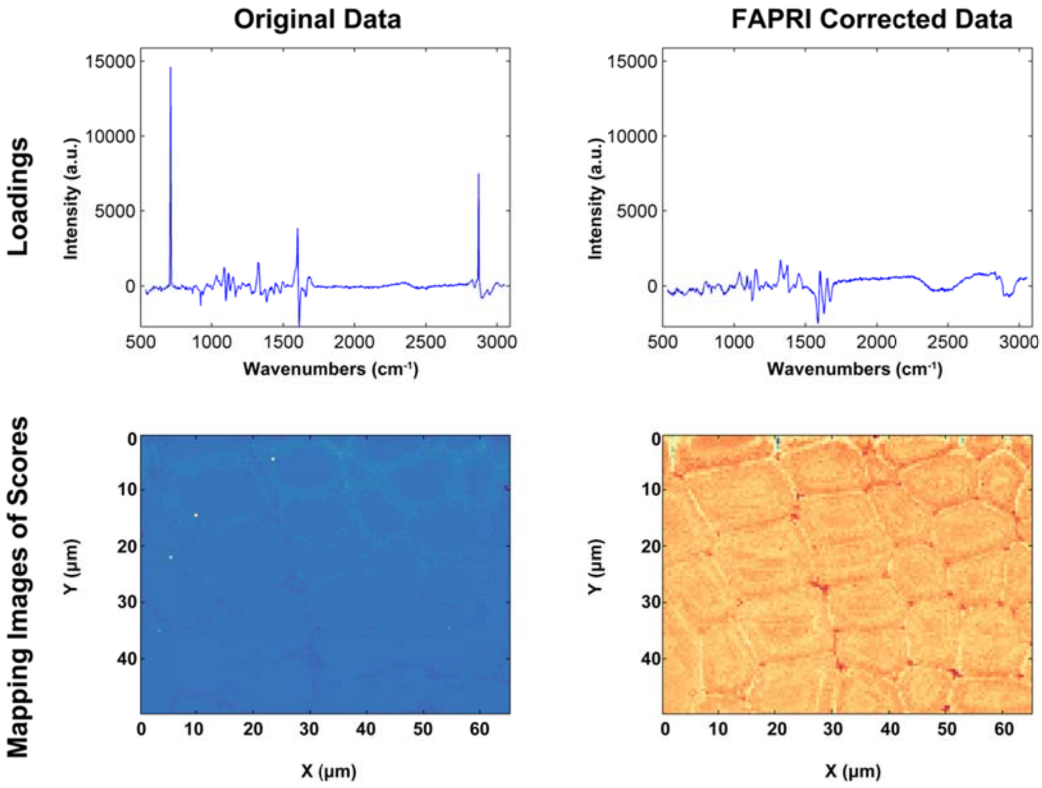

PCA results of original data and APRI corrected data (The 12th loading).

Figure S-18

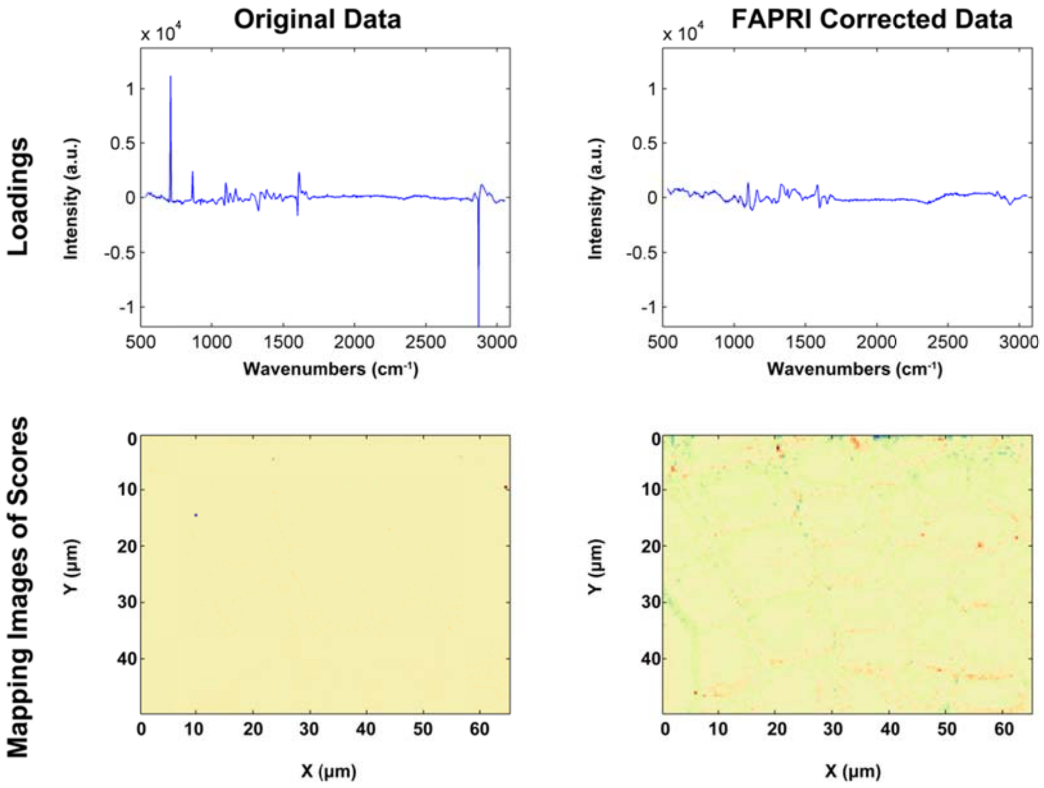

PCA results of original data and APRI corrected data (The 13th loading).

Figure S-19

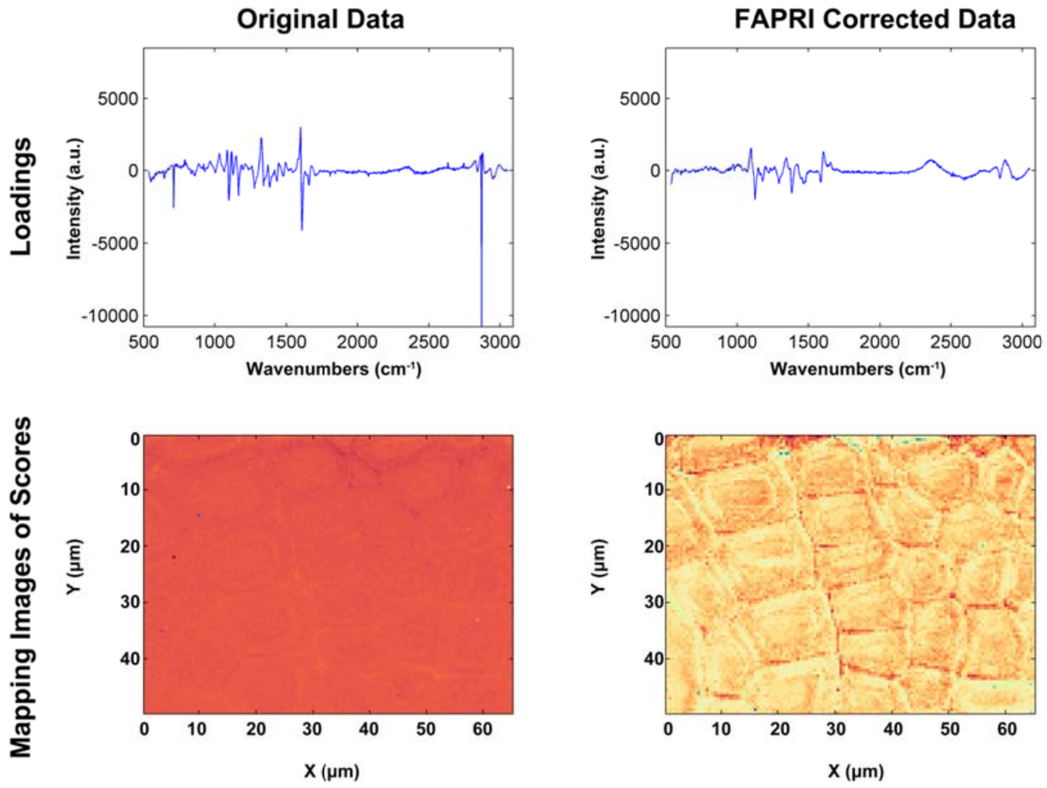

PCA results of original data and APRI corrected data (The 14th loading).

Figure S-20

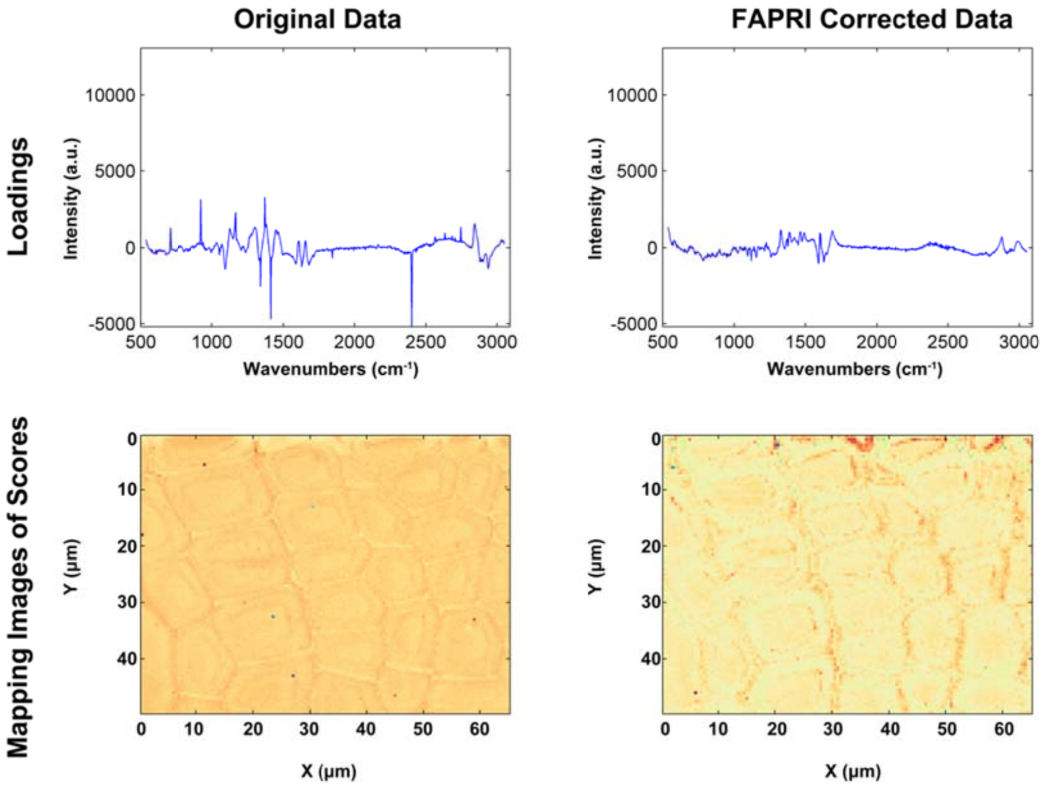

PCA results of original data and APRI corrected data (The 15th loading).

Figure S-21

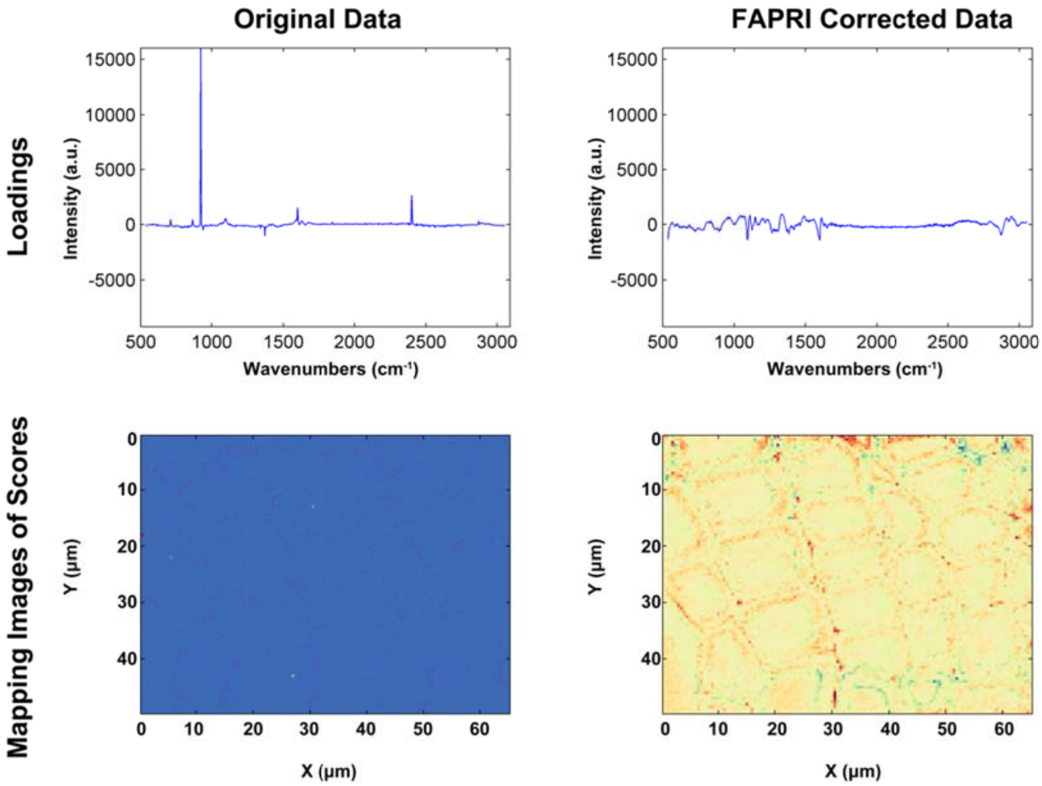

PCA results of original data and APRI corrected data (The 16th loading).

Figure S-22

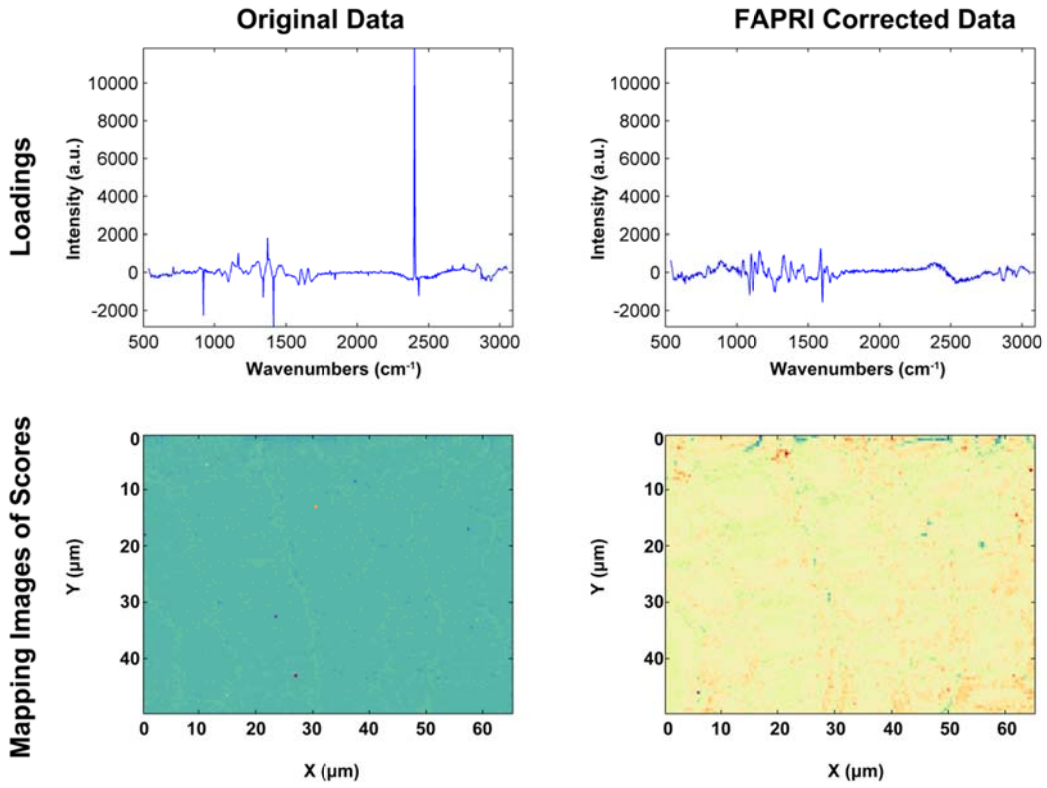

PCA results of original data and APRI corrected data (The 17th loading).

Figure S-23

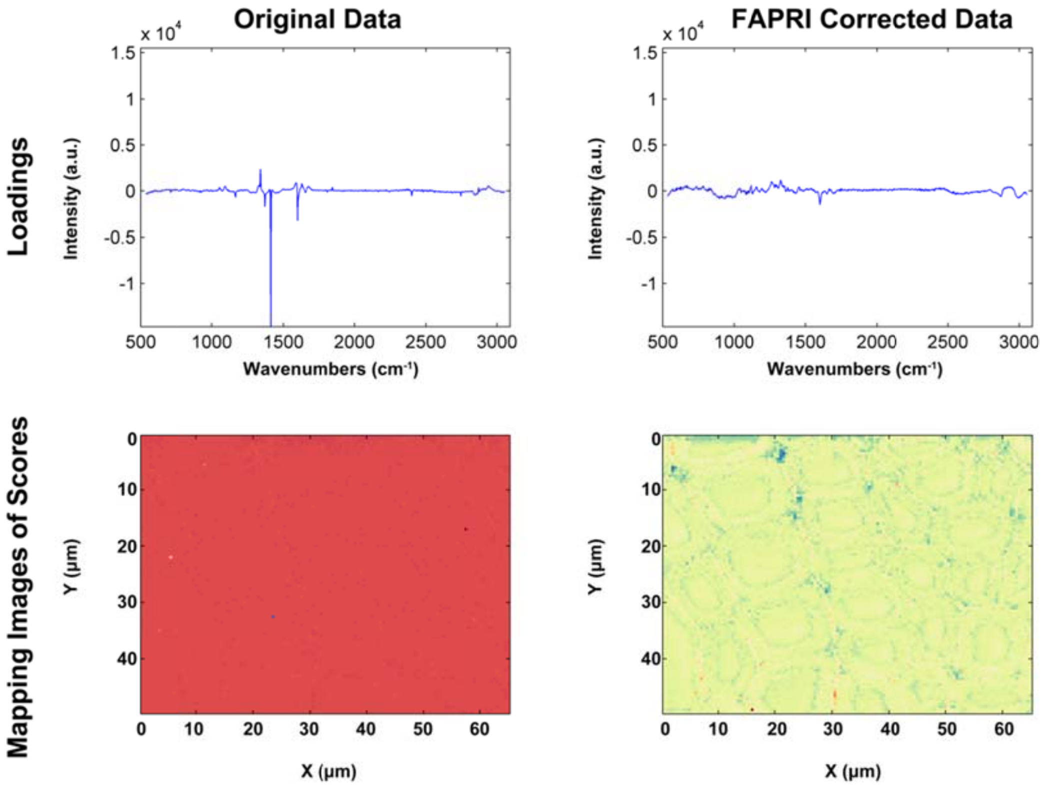

PCA results of original data and APRI corrected data (The 18th loading).

Figure S-24

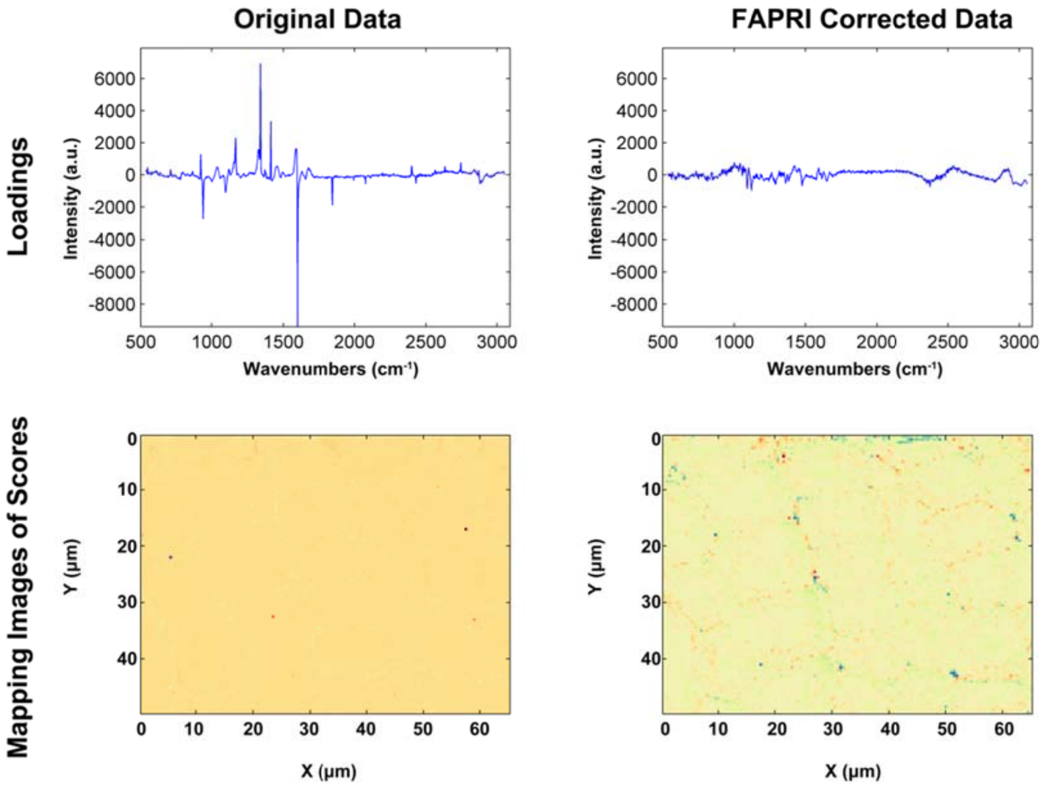

PCA results of original data and APRI corrected data (The 19th loading).

Figure S-25

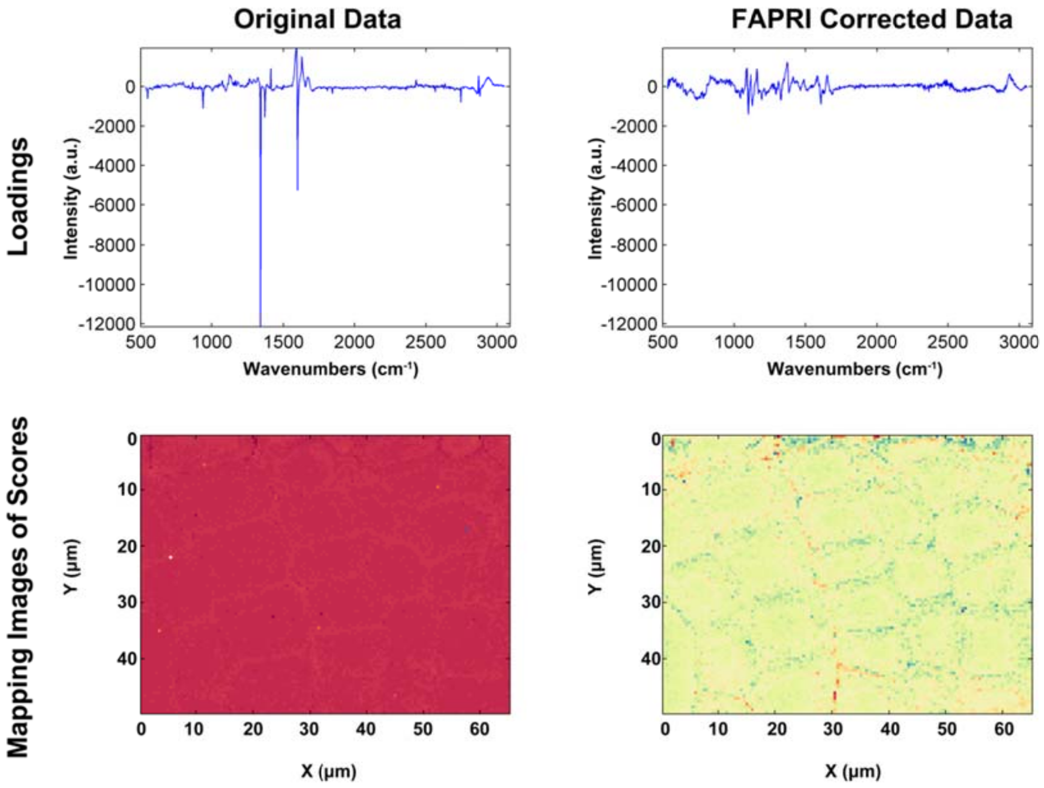

PCA results of original data and APRI corrected data (The 20th loading).
